# A Quantitative Relationship between Signal Detection in Attention and Approach/Avoidance Behavior

**DOI:** 10.3389/fpsyg.2017.00122

**Published:** 2017-02-21

**Authors:** Vijay Viswanathan, John P. Sheppard, Byoung W. Kim, Christopher L. Plantz, Hao Ying, Myung J. Lee, Kalyan Raman, Frank J. Mulhern, Martin P. Block, Bobby Calder, Sang Lee, Dale T. Mortensen, Anne J. Blood, Hans C. Breiter

**Affiliations:** ^1^Medill Integrated Marketing Communications, Northwestern UniversityEvanston, IL, USA; ^2^Applied Neuromarketing Consortium, Medill, Kellogg, and Feinberg Schools, Northwestern UniversityEvanston, IL, USA; ^3^Warren Wright Adolescent Center, Department of Psychiatry and Behavioral Sciences, Northwestern University Feinberg School of MedicineChicago, IL, USA; ^4^Laboratory of Neuroimaging and Genetics, and Mood and Motor Control Laboratory, Department of Psychiatry, Massachusetts General Hospital and Harvard Medical SchoolBoston, MA, USA; ^5^MGH Center for Translational Research in Prescription Drug Abuse, Department of Anesthesia, Massachusetts General Hospital and Harvard Medical SchoolBoston, MA, USA; ^6^Northwestern University and Massachusetts General Hospital Phenotype Genotype Project in Addiction and Mood DisordersChicago, IL, USA; ^7^Department of Materials Science and Engineering, University of Illinois at Urbana-ChampaignUrbana, IL, USA; ^8^Department of Electrical Engineering, Wayne State UniversityDetroit, MI, USA; ^9^Department of Marketing, Kellogg School of Management, Northwestern UniversityEvanston, IL, USA; ^10^Department of Economics, Northwestern University College of Arts and SciencesEvanston, IL, USA

**Keywords:** attention, reward, relative preference, signal detection theory, psychophysics, iterative modeling, neuroeconomics

## Abstract

This study examines how the domains of reward and attention, which are often studied as independent processes, in fact interact at a systems level. We operationalize divided attention with a continuous performance task and variables from signal detection theory (SDT), and reward/aversion with a keypress task measuring approach/avoidance in the framework of relative preference theory (RPT). Independent experiments with the same subjects showed a significant association between one SDT and two RPT variables, visualized as a three-dimensional structure. Holding one of these three variables constant, further showed a significant relationship between a loss aversion-like metric from the approach/avoidance task, and the response bias observed during the divided attention task. These results indicate that a more liberal response bias under signal detection (i.e., a higher tolerance for noise, resulting in a greater proportion of false alarms) is associated with higher “loss aversion.” Furthermore, our functional model suggests a mechanism for processing constraints with divided attention and reward/aversion. Together, our results argue for a systematic relationship between divided attention and reward/aversion processing in humans.

## Introduction

The association of attention and value-based choice is an important topic in behavioral neuroscience that, until relatively recently (e.g., Maunsell, 2004; Taylor et al., 2004; Small et al., 2005; Engelmann and Pessoa, 2007; Engelmann et al., 2009; Navalpakkam et al., 2009; Lim et al., 2011), has been often overlooked. Recent behavioral (Engelmann and Pessoa, 2007) and imaging (Taylor et al., 2004; Small et al., 2005; Engelmann et al., 2009; Lim et al., 2011) studies have begun to identify important relationships between reward and subjects' performance on attention or working memory tasks, and have also begun to identify brain areas whose activity is modulated by these relationships. Multiple studies have shown that task performance tends to improve as monetary incentives are increased, either in terms of increased detection sensitivity (Taylor et al., 2004; Engelmann and Pessoa, 2007; Engelmann et al., 2009) or faster reaction times (Small et al., 2005). Furthermore, changes in reward schedules affecting the gains and losses incurred for correct responses and false alarms cause subjects to systematically adjust their response biases in order to optimize reward on the task (Taylor et al., 2004).

Imaging studies have generally converged on a core frontoparietal network (widely implicated in attention; see Ptak, 2012), whose activation is increased during conditions of higher motivation (i.e., increased monetary incentives). Equally intriguing, spatial attention has been found to influence how subjects assign value to objects in a choice task (Lim et al., 2011), demonstrating that attention also affects value-based choice. In this task, ventromedial prefrontal cortex and ventral striatum were found to encode relative value signals indicating the difference in perceived value between the attended and unattended objects (Lim et al., 2011). Other evidence suggests similar neurotransmitters (e.g., dopamine) are involved with both attention and reward/aversion processing (Waelti et al., 2001). Psychopathology studies report alterations in attention with presumed disorders of reward processing, such as major depression or addiction, and addictive substances such as amphetamine can be used to treat attention deficit disorder (Biederman, 1992; Cook et al., 1995; Gossop et al., 2003).

While there is a body of evidence establishing that attention and reward are tightly coupled at the behavioral level and interact through common neural substrates, an open question is whether there are specific factors that can account for individual differences in both attention- and reward-related behaviors. We propose that a common integrated neurocognitive substrate may exist for the regulation of attention and processing of reward. Characteristics of subjects' performance on an attention task would thus be associated with aspects of their value-based choice behaviors when measured independently. The aforementioned studies do not address this question because they consider attention and reward as interacting components of a single task, and do not seek to identify the association of independent mathematical variables for these processes as a function that can be visualized. We sought to determine if there was a relationship between these variables that was mechanistic (i.e., observable as a mathematical structure in which the interplay of any two variables could be accurately quantified while holding the other variables constant).

Differentiating the contributions of attention- and reward-processing to behavior is also needed to better interpret results of a wide range of choice-based behavioral experiments (Kawagoe et al., 1998; Platt and Glimcher, 1999; see Maunsell, 2004 for review). In such paradigms, the experimenter alters the expected reward associated with making correct decisions pertaining to a presented stimulus, but in so doing the animal's attention to that stimulus is likely also affected; this raises the question of whether behavioral or neurophysiological effects observed in the experiment should be attributed to attention, reward, or both (Maunsell, 2004). Because these variables are so tightly coupled, it may be that the brain does not process reward and attention independently (e.g., Navalpakkam et al., 2009). Clearly, careful experiments that independently dissect reward vs. attention variables are in order to better assess the potential dependencies between these two domains.

Our approach in the present study therefore used independent experiments to isolate aspects of attention and reward behaviors in a common cohort of subjects. The tasks were completely independent so that any identified relationships could not be trivially explained by the use of a single common task, or to the use of identical experimental stimuli across the two tasks. These tasks included an approach/avoidance keypress task we have previously developed to gauge reward/aversion behavior (Kim et al., 2010), and a continuous performance task that assessed divided attention. The approach/avoidance task (Kim et al., 2010) gauged to what extent subjects would actively keypress to increase or decrease the amount of time they were exposed to face stimuli belonging to four categories: non-model male, non-model female, model male, and model female faces (Aharon et al., 2001). This validated task (Aharon et al., 2001; Elman et al., 2005; Strauss et al., 2005; Levy et al., 2008; Perlis et al., 2008; Gasic et al., 2009; Yamamoto et al., 2009; Kim et al., 2010; Viswanathan et al., 2015) quantified the effort subjects were willing to expend to approach or avoid each face stimulus. We then computed metrics that quantified the magnitude and predictability of the participants' keypress behavior. First, we calculated the mean numbers of keypresses subjects made to either approach (*K*^+^) or avoid (*K*^−^) face stimuli within each category. Second, we calculated the Shannon entropy (i.e., information; see Shannon and Weaver, 1949) of the distribution of keypress counts to approach (*H*^+^) or avoid (*H*^−^) the face stimuli within each category. Keypress measures of value such as those reported here have also been studied in the context of neuroimaging data, and have been specifically linked to activation of reward circuitry (Aharon et al., 2001; Strauss et al., 2005; Perlis et al., 2008; Gasic et al., 2009; Viswanathan et al., 2015) that appears affected by genotype (Perlis et al., 2008; Gasic et al., 2009). In addition, pattern variables such as Shannon entropy have been shown to be important metrics for quantifying neural processing (Viola et al., 1996; Rieke, 1997; Tiesinga et al., 2002; Reeke and Coop, 2004), and define the “information” that is processed in cognitive neuroscience (Breiter et al., 2006; Kim et al., 2010).

When graphed, K and H produce a value function resembling that of prospect theory (Kahneman and Tversky, 1979). In prospect theory, the objective value of an economic gain or loss is plotted on the x-axis against the subjective value assigned to that gain or loss by the subject on the y-axis. A key phenomenon observed through prospect theory is known as loss aversion which is trait-like: humans tend to be more averse to economic losses than they are to gains of the same magnitude, which results in a value function that is steeper for losses than for gains (Kahneman and Tversky, 1979; Tversky and Kahneman, 1992). Loss aversion can be calculated as the slope of the negative portion of the value function (involving losses) divided by the slope of the positive part of the curve. This computation can analogously be performed for the keypress data in our valuation graph using relative preference theory (RPT) (Kim et al., 2010), raising the question of whether attention-related metrics are related to this RPT variant of loss aversion across individual subjects.

To assess participants' divided attention, we used a continuous performance task (CPT), one of the most widely used neuropsychological tasks for this purpose (Beck et al., 1956; Davies and Parasuraman, 1982; Park and Waldman, 2014). During the CPT, participants viewed a continuous (sequential) presentation of letters, and responded to designated targets. Specifically, we employed an AX-CPT, which requires subjects to respond to the target (e.g., the letter “X”) only when preceded by a specific cue (e.g., the letter “A”) (Halperin et al., 1988; Seidman et al., 1998; Cohen et al., 1999). Performance on this task has classically been assessed using signal detection theory (SDT; Tanner and Swets, 1954; Green and Swets, 1966; McNicol, 1972; Verghese, 2001; Smith et al., 2013). By applying SDT, AX-CPTs allow the quantification of two key measures: sensitivity (d′) and response bias (β) (McNicol, 1972). The signal detection framework has received widespread acceptance and use within cognitive neuroscience (e.g., Eckstein et al., 2000; Verghese, 2001; Christensen et al., 2014; Park and Waldman, 2014).

We used an iterative modeling approach (Banks and Tran, 2009) to determine if any quantitative relationships existed between features of reward behavior (*K*^+^ and *K*^−^ as *K*^±^, *H*^+^ and *H*^−^ as *H*^±^) and attention (β, d′) from these two tasks. To foreshadow our results, our main findings included a significant relationship between the response bias (β) on the signal detection task and the ***K*** and ***H*** measures from the approach/avoidance task. No significant relationships were observed between the sensitivity ***d***′ during divided attention and the ***K*** and ***H*** approach/avoidance measures. In assessing the mathematical model between **K**, **H**, and **β** across subjects, smaller β (i.e., more liberal response bias) during divided attention was associated with higher predictability of avoidance keypressing and lower predictability of approach keypressing during the approach/avoidance task (e.g., analogous to having more loss aversion). As presented in several talks and meetings (e.g., Breiter, 2012, 2014; Raman, 2013), our study provides empirical evidence for a quantitative link between divided attention and reward/aversion behaviors assessed though independent tasks.

## Methods

### Subjects

All subjects were recruited by advertisements from the New England region. As described for other papers using subjects from the MGH Phenotype Genotype Project (e.g., Strauss et al., 2005; Perlis et al., 2008; Gasic et al., 2009; Kim et al., 2010; Viswanathan et al., 2015), subject recruitment stopped after a set temporal window for recruitment, where target recruitment for healthy controls sought 50–100 subjects. This resulted in 77 subjects meeting criteria to be considered healthy controls, meaning that they were medically healthy and without mental illness, and they were neither family members of a participant with cocaine dependence or polysubstance abuse, nor a participant with major depressive disorder. Of these 77 subjects, 6 subjects did not complete any divided attention task and another 6 subjects completed an earlier version of the divided attention task than the one reported in this manuscript, resulting in the exclusion of these 12 subjects. Data from the 6 subjects who completed the earlier divided attention task could not be used because there was a very small number of false cues and false targets on this version of the task, which limited the ability to compute accurate false alarm rates or signal detection metrics. Of the remaining 65 subjects who completed the reported version of the divided attention task, 18 additional subjects were excluded because they had false alarm rates of 0, making it impossible to compute valid signal detection metrics (i.e., d′ and β). This resulted in a final cohort of 47 subjects who had valid data from both the divided attention and reward/aversion (beauty keypress) tasks. These 47 subjects comprise the cohort whose data are reported in the present study; their **K**, **H** keypress data alone (from the reward/aversion task) was previously reported within the larger group of 77 subjects by Kim et al. (2010). For these subjects, the mean age was 32.0 ± 10.6 years (SD), mean educational history was 15.8 ± 2.9 years, and subjects were 22 of 47 (46.8%) female, and 41 of 47 (87.2%) right-handed, with the following race identification: 34 of 47 European-American, 2 of 47 Native American, 7 of 47 African-American, and 4 of 47 Asian. All subjects underwent a clinical interview with a psychiatrist that included the Structured Clinical Interview for Diagnosis—Axis I (SCID-I/P; First et al., 1996). Race was determined by individual self-identification on a standardized form (Benson and Marano, 1998), and handedness via the Edinburgh Handedness Inventory (Oldfield, 1971). Eligible subjects were age 19–54, without any current or lifetime DSM-IV Axis I disorder or major medical illness known to influence brain structure or function, including neurologic disease, HIV, or Hepatitis C as determined by assay. Medical illness was assessed via a physician-led review of body systems and a physical exam. Female subjects were studied during their mid-follicular phase based upon self-reported menstrual history, with confirmation at the time of testing based on hormonal testing with a urine assay. All subjects were studied at normal or corrected-to-normal vision.

### Ethics statement

All subjects signed written informed consent prior to participation, approved for this study by the Institutional Review Board of Massachusetts General Hospital (i.e., Partners Human Research Committee, Partners Healthcare), and all experiments were conducted in accordance with the principles of the Declaration of Helsinki.

### Experimental paradigms

The divided attention and approach/avoidance tasks were conducted alongside multiple other procedures as part of the MGH Phenotype Genotype Project (PGP). Following accepted methods, all data collection was counterbalanced within the fMRI environment and outside of it. None of the MRI data from the two experiments described herein has been published. Please see the Supplemental Information section for a complete description of data acquired by the PGP.

#### Approach/avoidance task

**Figure 1:**
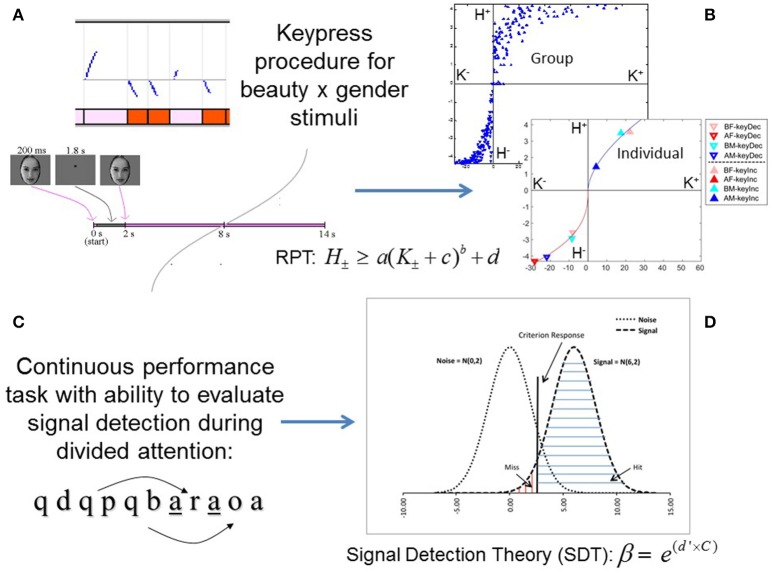
**Experimental paradigms for approach/avoidance and signal detection tasks. (A)** A schema for the keypress paradigm shows, at top, raster plots of keypressing effects on face viewing time (y-axis) as blue curves going up or down from a default viewing time of 6 s. Pink and red blocks represent the presentation of beautiful and average female faces, respectively. Below the raster plots, the timing of face presentation on each trial is schematized (Methods). **(B)** Keypressing data yielded distinct boundary envelopes to the (***K***, ***H***) value functions at the group level, and were well fit by power-law functions in individual subjects, when plotting mean keypresses (K) against the Shannon entropy of keypress responses (H, information) across face categories (Methods). **(C)** A visual continuous performance task quantified signal detection parameters during divided attention. One letter was shown per second in the center of the visual field. Subjects responded when a target letter (“a”) appeared exactly four letters after the cue (“q”). Cue-target pairs could be interleaved, necessitating divided attention. **(D)** Signal detection analysis allowed estimation of a criterion response, signal and noise distributions, as well as β and d′ variables for the continuous performance task (Methods).

An approach/avoidance keypress task was used to determine each subject's relative preference toward an ensemble of faces presented on a screen (Kim et al., 2010). The keypress experiment allowed rapid responses in which subjects were able to press different sets of keys in order to change the viewing duration for each presented stimulus (Aharon et al., 2001; Kim et al., 2010). If subjects disliked a picture stimulus and wanted it to disappear faster, they could alternate pressing one set of keys (#3 and #4 on the button box), whereas if they liked a picture stimulus and wanted it to remain on the screen longer, they could alternate pressing another set of keys (#1 and #2 on the button box). Subjects therefore had the choice to do nothing (default condition), increase their viewing time (i.e., “approach”), decrease their viewing time (i.e., “avoid”), or a combination of both increasing and decreasing the stimulus viewing time (Figure 1A). A slider was displayed to the left of each picture to indicate the total remaining viewing time. Presented faces fell within the following experimental categories: beautiful (models) and average (non-models) faces of both genders [i.e., beautiful female (BF), average female (AF), beautiful male (BM), and average male (AM); Aharon et al., 2001]. Each category of faces had 20 different faces in it. We used the same 80 faces with each subject. Importantly, the experiment used the same number of itemsets (i.e., categories of pictures) and items per itemset (i.e., number of pictures) for every subject. The itemset size sets the range of possible H values, but does not determine the specific H value, so it was important to keep the number of items (i.e., pictures) constant across subjects (Kim et al., 2010; Lee et al., 2015).

The keypress procedure was implemented with Matlab software. This task captured the reward valuation attributed to each observed face, and quantified positive (approach) and negative (avoidance) preferences involving (i) decision-making regarding the valence of behavior, and (ii) judgments that determine the magnitude of approach and avoidance (Kim et al., 2010; Lee et al., 2015). The objective was to determine how much effort each subject was willing to trade for viewing each facial expression relative to a default viewing time. Subjects were told that they would be exposed to a series of pictures that would change every 8 s (Figure 1A) if they pressed no keys. As published previously (Aharon et al., 2001; Elman et al., 2005; Strauss et al., 2005; Levy et al., 2008; Makris et al., 2008; Perlis et al., 2008; Gasic et al., 2009; Yamamoto et al., 2009; Kim et al., 2010; Lee et al., 2015; Viswanathan et al., 2015), each experimental stimulus would be initially presented for 0.2 s and replaced by a fixation point for 1.8 s (the “decision block”), until the face reappeared at 2 s and the subject was given the option to increase or decrease the viewing time via keypressing the “judgment block”). The relationship between the number of keypresses made by the subject to approach or avoid the face stimuli and the updated viewing time followed previous methods, and utilized the following resistive function (Aharon et al., 2001; Elman et al., 2005; Strauss et al., 2005; Levy et al., 2008; Makris et al., 2008; Perlis et al., 2008; Gasic et al., 2009; Yamamoto et al., 2009; Kim et al., 2010; Lee et al., 2015; Viswanathan et al., 2015):

(1) $$t_{n}=\sum_{n=1}^{N}t_{n\;-\;1}+(A-t_{n\;-\;1})/J,$$

where ***t_n_*** is the updated viewing time achieved after the keypress, ***t*_*n* - 1_** is the allotted viewing time prior to the keypress, ***A*** is equal to 0 s for avoidance keypresses reducing the viewing time or 14 s for approach keypresses increasing the viewing time, and *J* is a scaling constant equal to 40. The default viewing time, ***t***_**0**_, was equal to 6 s. The purpose of the resistive function was twofold: (a) to sequentially increase the effort needed for changing viewing time and the value thereof, and (b) to implement limits as to how much shorter or longer the stimulus duration could be modulated at maximal keypressing (so that the stimulus duration could not be indefinitely extended) (Aharon et al., 2001; Kim et al., 2010). The resistive function accomplishes both of these because with each subsequent keypress, the magnitude of the resulting change in viewing time diminishes relative to the previous change. This maintains viewer engagement by progressively increasing the effort (Walton et al., 2006, 2007; Croxson et al., 2009) involved with this intrinsic motivation-based judgment (Deci and Ryan, 1985; Bandura, 1997), and creates consistent bounds on the range of possible viewing times. Our choice of constants in the resistive function (i.e., *A, J*, and ***t***_**0**_) resulted in stimulus viewing durations that could theoretically range from 0 s (maximum avoidance keypressing) to 12 s (maximum approach keypressing) (Kim et al., 2010), where the 12 s keypressing interval followed a 2 s decision phase in the task (Figure 1A).

Subjects were told this with the goal of preventing them from keypressing simply to shorten the length of time required to complete the experiment. In actuality, the exact same number of total stimuli was presented to each subject regardless of their keypress behavior, entailing that the experiment could vary somewhat in overall duration depending on subjects' keypress behavior across all stimuli.

#### Divided attention task

Subjects performed a continuous performance task [CPT; (Beck et al., 1956; Davies and Parasuraman, 1982) using visual stimuli (Seidman et al., 1998). In this task, subjects were required to respond to an “A” (target) following a “Q” (cue) after three intervening letters. This task added interference and divided attention load by, respectively, including false cues and/or false targets, and by intermingling a subset of QxyzA sequences within each other (e.g., QxQyAzA) (Figure 1C). Each letter was presented for 200 ms and followed by a fixation point for 800 ms. The task was administered as three blocks, with each block lasting 60 s. Subjects were instructed to respond to targets with a button press but not to respond to non-targets. Following a signal detection framework (Tanner and Swets, 1954; Green and Swets, 1966; McNicol, 1972; Verghese, 2001; Smith et al., 2013), hits, misses, correct rejections, and false alarms were assessed and used in statistical analyses. Hits indicate correct responses to cues, misses indicate failures to respond to cues, correct rejections indicate the correct absence of a response to a non-cue, and false alarms indicate erroneous responses to non-cues (Figure 1D).

### Data analysis

a. Computation of Reward and Attention Measures

### Approach/avoidance task

#### Descriptive statistical measures

Descriptive statistics were used to summarize subjects' keypress responses. The central relationship we considered was that between the mean numbers of keypresses to approach or avoid, averaged across all stimuli within each category of faces (***K***^+^ and ***K***^−^), and the Shannon entropy of the approach/avoidance keypresses counts within each face category (***H***^+^ and ***H***^−^). To do this, we separately computed each subject's mean number of keypress responses to either approach (***K***^+^) or avoid (***K***^−^) stimuli within each face category. Next, we computed the Shannon entropy (Shannon and Weaver, 1949) describing subjects' patterns of approach/avoidance keypressing (Kim et al., 2010) for the positive (***H***^+^) and negative (***H***^−^) keypress responses across each face category. It should be noted that prior work (Aharon et al., 2001) showed a dissociation between ratings of esthetic attractiveness of stimuli (consistent with individuals in pictures being models or not) and keypress results controlling the length of viewing. Namely, the mean keypress measure K and the preference predictability measure H, can differ markedly from the perceived attractiveness metric at the core of an individual being a model or not.

We used the following approach to compute Shannon entropy separately for the positive (approach) and negative (avoidance) keypress responses in each category. First, consider an ensemble of keypress responses (i.e., numbers of keypresses) ***A*** across stimuli within a single face category: ***A***^**±**^ = (***a***_1_, ***a***_2_, …, ***a***_*n*_). We can then define the relative proportions of the keypress responses for the individual stimuli, ***p***_*i*_ (Kim et al., 2010), such that:

(2) $$p_{i}=a_{i}/\sum_{j\;=\;1}^{n}a_{j}.$$

Using these proportions of the keypress responses, the Shannon entropy of the keypress response pattern can be computed for an individual face category as follows:

(3) $$H^{\pm }=\sum_{i\;=\;1}^{n}p_{i}\;\log_{2}\frac{1}{p_{i}}$$

Table 1 provides summary statistics of K^+^, K^−^, H^+^ and H^−^ averaged across subjects and face categories. Observations with a mean keypress (*K*^**±**^) of 0 were excluded from the analysis. A mean keypress (**K^±^**) of 0 for a category implies that the entire ensemble of stimuli in that category all had zero keypresses; the resulting proportions *p*_*i*_ are therefore undefined (i.e., since 0/0 is undefined) and the entropy (***H***^**±**^) cannot be computed in such cases. Across all 47 subjects in the final cohort, there were 13 observations with a K^−^ of 0 (from 9 subjects) and 91 observations with a K^+^ of 0 (from 42 subjects). Recall that *K* and *H* variables were computed within each subject for each individual face category. Overall, for the models computed across all subjects of the form H = f(K, β), we ended up with 138 total valid data points for the “approach” models using H^+^ as the dependent variable, and 181 valid data points for “avoidance” models using H^−^ as the dependent variable. No exclusion of data points (i.e., exclusion of specific *K* and *H* metrics for individual face categories) led to the complete exclusion of a participant's (*K*^±^, *H*^±^) data from the analysis.

**Table 1:** **Descriptive statistics for K, H, β, d′, predictability bias (PB), and false alarm rate (FA)**.

**Variable**	***N***	**Mean**	***SD***	**Minimum**	**Maximum**
β	47	2.57	1.26	0.73	5.34
d′	47	2.48	0.68	0.77	3.64
FA	47	0.062	0.041	0.029	0.206
PB	41	2.26	1.79	0.00	7.74
K^+^	138	16.84	20.01	0.05	86.95
K^−^	181	11.37	7.81	0.15	29.30
H^+^	138	2.34	1.46	0.00	4.24
H^−^	181	3.28	1.23	0.00	4.32

*N is the total number of observations used to compute each descriptive statistic, and SD is the standard deviation. The minimum and maximum values provide information on the observed range for each variable*.

#### Computing relative preference theory structure

After computing the values of ***K***^**±**^ and ***H***^**±**^ for each face category, (***K***, ***H***) functions were generated by plotting the Shannon entropy ***H***^**±**^ against the mean keypresses ***K***^**±**^ for all face categories in an individual subject; we refer to this function as the (***K***, ***H***) value function. (***K***, ***H***) data can also be plotted across multiple subjects to visualize data at the group level. At the group level, we confirmed that (***K***, ***H***) data contained boundary envelopes that conformed well to power-law functions (***H*** = ***a K***^*b*^; see edges of group data in Figure 1B). In previous work, we have also reported logarithmic fits to the (***K***, ***H***) value functions, as well as logarithmic fits to boundary envelopes containing group (***K***, ***H***) data (Kim et al., 2010). At the individual subject level, we also fit simple power-law (***H*** = ***a K***^*b*^) functions to the (***K***, ***H***) data for approach and avoidance across face categories within individual subjects (e.g., Figure 1B). The fits for power-law functions were obtained by performing simple linear regression of ***ln H*** on ***ln K***.

After defining the (***K***, ***H***) valuation graphs, we were also able to compute how subjects tended to respond to face stimuli when they were aversive compared to when they were attractive. This was done by comparing the slopes of the valuation graph for the negative (avoidance) segment to the positive (approach) segment. Comparing the slopes in this way follows methods previously described under Prospect Theory by Kahneman and Tversky (1979) and Tversky and Kahneman (1991), but has a different interpretation when used in the context of relative preference theory. For the value function described under prospect theory, the objective value of economic gains or losses is plotted on the x-axis against the subjective value of the gains or losses to the individual on the y-axis. The absolute value of the ratio of the slope of the negative value function (*s*^−^) to the positive segment of the value function slope (*s*^+^) is then referred to as *loss aversion*: *LA* = |*s*^−^/*s*^+^|, describing the extent to which subjects overweight losses relative to gains of equal magnitude (Tversky and Kahneman, 1991; Köbberling and Wakker, 2005; Schmidt and Zank, 2005).

Analogously, we applied a local definition of loss aversion (Benartzi and Thaler, 1995; Abdellaoui et al., 2007; Booij and van de Kuilen, 2009) to our (***K***, ***H***) valuation graphs for each individual subject to define *predictability bias* (PB) in the framework of RPT. Predictability bias indicates how much more predictable subjects' preference patterns are during avoidance (aversion) behaviors relative to approach (reward) behaviors. To compute predictability bias, *s*^−^ and *s*^+^ were computed as the average of the slopes of the negative and positive components of the (***K***, ***H***) function over the 10% of the curves closest to the origin:

(4) $$PB=\left|\frac{s^{-}}{s^{+}}\right|=\left|\frac{\int_{-x_{c}}^{0}f_{-}^{\prime }(x)dx/x_{c}}{\int_{0}^{x_{c}}f_{+}^{\prime }(x)dx/x_{c}}\right|,$$

where $\;f_{+}^{\prime }\left(x\right)$ and $\;f_{-}^{\prime }\left(x\right)$ are the first derivatives of the positive (approach) and negative (avoidance) components of the (***K***, ***H***) valuation graph, respectively, and *x*_*c*_ represents the range of the valuation function over which the slopes are averaged; namely, *x*_*c*_ covers the range of K values that span up to 10% of the maximum absolute *H*^±^ value observed on a subject-wise basis.

### Divided attention task

For analyzing data from the divided attention task, classic signal detection measures (β and d′) were computed after first computing the hit and false alarm rates from the task (Green and Swets, 1966; McNicol, 1972; Verghese, 2001). Hit rates were computed as the ratio of the number of correct responses for identifying targets to the total number of (true) targets. False alarm rates were calculated as the ratio of the number of erroneous responses to non-targets to the total number of non-targets (i.e., distractors). With these hit and false alarm rates, we then computed β (beta) and d′ (d-prime) following standard signal detection methods (Table 1). d′ was computed as follows:

(5) d′=Z(hit rate)−Z(false alarm rate),

where *Z(p)* is the inverse of the cumulative distribution function of the standard normal distribution (i.e., $Z\left(p\right)=\sqrt{2}erf^{-1}\left(2p-1\right),\;p\in \left[0,1\right],\;$ and *erf* is the error function). Beta is defined as follows:

(6) $$\beta =e^{(Z{\left(false\;alarm\;rate\right)}^{2}-Z{\left(hit\;rate\right)}^{2})/2}.$$

b. Relationships between RPT and SDT Variables

The aims of our paper were to (1) test for a mathematical relationship between variables related to reward/aversion (obtained from our relative preference theory (RPT) keypress task), and variables related to divided attention (obtained from the divided attention task) within the same cohort of subjects, and (2) determine if this mathematical model allowed the interplay of any two variables to be accurately quantified while holding the others constant. To accomplish this, we fit a series of mathematical models in which the RPT (reward/aversion) variables ***K*** and ***H*** were related to the divided attention variables β or d′, and then assessed if the observed mathematical model provided interpretive insight in the quantitative interplay between variables. For the first aim, we tested models formulated such that ***H*** was defined as a function of ***K*** and β or of ***K*** and d′ (i.e., we fit functions of the form ***H*** = f(***K***, β) or ***H*** = f(***K***, d′)). Three model formulations were evaluated (examples below show the models for ***H*** = f(***K***, β); for the models of the form ***H*** = f(***K***,d′), d′ is substituted for β):

1. Logarithmic model: H = log a + b log β + clog K
2. Multiplicative power-law: H = aβ^b^K^c^
3. Additive power-law: H = a + β^b^ + K^c^.

In the above equations, *a, b*, and *c* are coefficients fit separately for each of the three models.

To assess the strengths of the fits for these three models with H, K and β, or H, K, and d′, we computed Root Mean Square Error (RMSE) for each model. RMSE was computed as follows: $\;RMSE=\sqrt{\frac{\sum_{i=1}^{n}{\left({Ĥ}_{i}-H_{i}\right)}^{2}}{n}}$, where Ĥ_*i*_ is the value of the entropy *H* (i.e., the dependent variable) predicted by the model, and *H*_*i*_ is the observed (actual) value of the entropy for face category *i*. We also computed R, where R is the Coefficient of Determination and is the square root of the ratio of Regression Sum of Squares (SSR) to Total Sum of Squares (SST). While SSR is a measure of the variation of the predicted *H* values around the mean observed *H* value, SST is a measure of the variation of the actual observed *H* values around the mean. Therefore, *R* = $\sqrt{\frac{\sum_{i=1}^{n}{\left({Ĥ}_{i}-\overset{-}{H}\right)}^{2}}{\sum_{i=1}^{n}{\left(H_{i}-\overset{-}{H}\right)}^{2}}}$, where Ĥ_*i*_ is again the predicted entropy and *H*_*i*_ is the observed entropy for category *i*, and $\overset{-}{H}$ is the average entropy across all face categories.

After assessing the goodness of fits for the three different models that related the *H, K*, and β, d′ variables, we sought as a second aim to this study to evaluate a potential relationship between β or d′ from the divided attention task and predictability bias (i.e., the loss aversion metric) as determined from the approach/avoidance task. For example, using a potential relationship between K, H, and the response bias β, we first approached this question analytically by examining the multiplicative power-law formulation relating H, K, and β: H = aβ^b^K^c^. By inserting the estimated model coefficients and using the mean values of K observed across stimuli and subjects (Results), we obtained quantitative model predictions for the average values of H for approach (H^+^) and avoidance (H^−^) expected as a function of β (**Figure 4A**) as follows:

1. Given the power law formulation H = a β^b^ K^c^, use the estimated coefficients of *b* and *c* from the approach (***H***^+^) and avoidance (***H***^−^) models for predicting approach (***H***^+^) and avoidance (***H***^−^) entropy, respectively (Table 2).
2. Use the coefficient *a* estimated from the approach (***H***^+^) model as a common coefficient for predicting both approach (***H***^+^) and avoidance (***H***^−^) entropy (i.e., *a* = 1.066; see Table 2). Note that the coefficient *a* is simply a scaling constant in the multiplicative power law model; this coefficient was kept constant between the approach and avoidance models in order to allow for a direct relative comparison of approach (***H***^+^) and avoidance (***H***^−^) entropy as a function of β.
3. For predicting both approach (***H***^+^) and avoidance (***H***^−^) entropy, keep ***K*** constant at the mean level of ***K***^+^ observed across subjects (i.e., K = 16.84; see Table 1). Using the same value of K for both the approach (***K***^+^) and avoidance (***K***^−^) models allows for a direct comparison of the approach (***H***^+^) and avoidance (***H***^−^) entropy as a function of β.
4. Finally, vary β in small steps over the range [0, 6] to predict ***H***^+^ and ***H***^−^ across a range of potential β values.

**Table 2:** **Fits for different models of the form H = f(K,β)**.

**Approach (H+)**	**Parameter**	**Logarithmic: H = a + b·lnβ + c·ln K**	Power Law Multiplicative: H = a * (β)^b^ * (K)^c^	**Power Law Additive: H = a + β^b^ + K^c^**
		**Estimate**	**Estimate**	**Estimate**
Intercept/scaling constant	a	0.847	1.066	−1.112
		[0.535, 1.160]	[0.865, 1.268]	[−1.405, −0.819]
		*t*_(135)_ = 5.36	*t*_(135)_ = 10.45	*t*_(135)_ = −7.51
		*p* = 3.42e-7	*p* = 4.25e-19	*p* = 7.32e-12
β	b	0.321	0.092	0.240
		[0.042, 0.600]	[−0.013, 0.196]	[0.046, 0.434]
		*t*_(135)_ = 2.28	*t*_(135)_ = 1.73	*t*_(135)_ = 2.44
		*p* = 0.024	*p* = 0.085	*p* = 0.016
		*q* = 0.0304	*q* = 0.0557	*q* = 0.0304
K^+^	c	0.667	0.319	0.349
		[0.582, 0.751]	[0.267, 0.370]	[0.324, 0.374]
		*t*_(135)_ = 15.61	*t*_(135)_ = 12.30	*t*_(135)_ = 27.36
		*p* = 5.10e-32	*p* = 8.81e-24	*p* = 6.33e-57
RMSE		0.8682	0.8667	0.8675
R		0.8079	0.8086	0.8082
Model F-stat		*F*_(2, 135)_ = 127	*F*_(2, 135)_ = 421	*F*_(2, 135)_ = 127
Model sig.		*p* = 9.94e-32	*p* = 2.53e-68	*p* = 8.97e-32
**Avoidance (H**−**)**	**Parameter**	**Estimate**	**Estimate**	**Estimate**
Intercept/scaling constant	a	1.230	1.523	−0.540
		[1.031, 1.430]	[1.369, 1.678]	[−0.756, −0.324]
		*t*_(178)_ = 12.16	*t*_(178)_ = 19.48	*t*_(178)_ = −4.94
		*p* = 3.65e-25	*p* = 5.35e-46	*p* = 1.81e-6
β	b	0.196	0.044	0.141
		[0.052, 0.340]	[−0.003, 0.090]	[0.006, 0.277]
		*t*_(178)_ = 2.69	*t*_(178)_ = 1.86	*t*_(178)_ = 2.06
		*p* = 0.008	*p* = 0.065	*p* = 0.041
		*q* = 0.0304	*q* = 0.0497	*q* = 0.0376
K^−^	c	0.951	0.337	0.443
		[0.887, 1.014]	[0.303, 0.371]	[0.423, 0.463]
		*t*_(178)_ = 29.4	*t*_(178)_ = 19.32	*t*_(178)_ = 43.43
		*p* = 2.70e-70	*p* = 1.44e-45	*p* = 1.17e-96
RMSE		0.5108	0.5656	0.5930
R		0.9112	0.8899	0.8782
Model F-stat		*F*_(2, 178)_ = 436	*F*_(2, 178)_ = 2250	*F*_(2, 178)_ = 300
Model sig.		*p* = 2.74e-69	*p* = 2.92e-141	*p* = 9.43e-58

*95% confidence intervals are in brackets. RMSE and R are measures of model fit as described in Table 3. FDR-adjusted q values are also provided for the b coefficient fits. These q values correct for the 6 total model comparisons made and indicate the minimum false discovery rate at which these coefficients may be declared significant*.

After using our fitted model to predict the effects of β on H^−^ and H^+^ (**Figure 4A**), we computed two proxy measures of the predictability bias: a ratio metric (H^−^/H^+^) and a difference metric (H^−^–H^+^) of the approach and avoidance entropies (**Figure 4B**). This allowed us to make quantitative predictions using our model of how variations in β could affect the predictability bias. The ratio metric (H^−^/H^+^) has traditionally been used in behavioral studies, although the difference metric has also been used previously the context of neuroimaging (Tom et al., 2007).

c. Multiple comparisons corrections

When drawing conclusions regarding the statistical significance of our modeling work relating the divided attention variables d′ and β to the reward/aversion variables ***K*** and ***H*** from our approach/avoidance keypress task, it is essential to keep in mind the large number of statistical tests that were performed, which increases the probability of incorrect rejections of the null hypothesis (i.e., Type I error). Our initial modeling involved univariate modeling of power-law relationships between ***K***, ***H***, β and d′. There were four types of univariate models (***K*** vs. β, ***H*** vs. β, ***K*** vs. d′, and ***H*** vs. d′), and two instances of each model since they were fit to both approach and avoidance keypress data. (Note that the significance of the power-law fits is equivalent regardless of which variable is the dependent variable, since we fit the models via linear regression after a log-log transformation.) Thus, in total, there were 8 statistical tests considered with the univariate data. For the multivariate modeling of the H = f(K, β) and H = f(K, d′) models, there were three different models considered in each case, fit separately to approach and avoidance data; this resulted in another 12 comparisons for the multivariate modeling. Therefore, in total, there were 20 statistical tests performed in our analysis of the relationship between divided attention and reward/aversion variables. Since Bonferroni correction is well known to be overly conservative, we chose to address these multiple comparison issues by applying false discovery rate (FDR) correction and examining the resulting *q*-values of our statistical tests (Benjamini and Hochberg, 1995; Storey, 2002). The *q*-values indicate the minimum false discovery rates that can be claimed when declaring the corresponding statistical tests significant, and the false discovery rate is the proportion of rejected null hypotheses that are false positives.

## Results

### Experimental tasks

#### Divided attention task

Subject performance on the divided attention task is summarized in Table 1, which provides average values and standard deviations of β, d′, and the false alarm rate observed across subjects.

#### Approach/avoidance task

**Figure 2:**
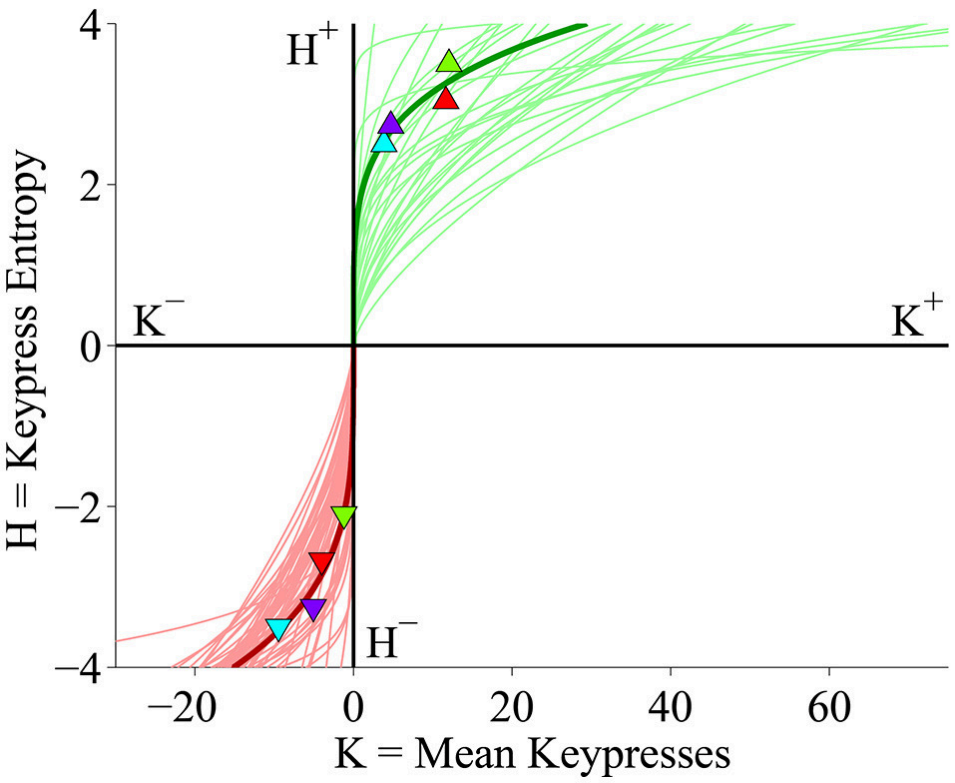
**(*K*, *H*) value functions display keypress behavior on the approach/avoidance beauty keypress task for all 47 subjects**. The x-axis indicates mean keypress intensity (***K***) for each face category in terms of the average number of keypresses to approach (right) or avoid (left) each category of faces. The y-axis indicates the Shannon entropy (***H***) of the ensemble of keypress responses to approach or avoid for each category of faces (Methods). Red and green traces indicate power-law fits to the (***K***, ***H***) data computed within each individual subject by performing linear regression of ***ln H*** against ***ln K***. Symbols and dark green and red traces indicate the (***K***, ***H***) data points and power-law fit (respectively) for a representative subject; light green and red traces indicate the power-law fits for the remaining subjects in the cohort.

For each individual subject, we plotted the values of *H* as a function of *K* across all face categories, resulting in (*K, H*) valuation graphs (Figures 1B, 2). Average values and standard deviations of *K*^±^ and *H*^±^ observed across subjects are presented in Table 1. The relationship is depicted in Figure 1B for both individual and group data, and in Figure 2 for individual subject data. The ***K, H*** relationship observed here has been described previously by Kim et al. (2010), and is the same in the current cohort as what was observed in the larger sample. In the current study as well as previous analyses of this data (Kim et al., 2010), we found that power-law functions of the form ***H*** = ***a K***^*b*^ offered a strong fit both to the (***K***, ***H***) value functions in individual subjects as well as to boundary envelopes containing the group (***K, H***) data (Figures 1B, 2). We observed significant power-law scaling for both approach (***H***^+^ = 1.381 ***K***^0.257^) and avoidance (***H***^−^ = 1.463 ***K***^0.369^) data (Supplementary Table S1; all *p* < 10^−21^ for exponential term). Figure 2 displays the power-law fits computed for all subjects in the cohort, and shows overlaid (***K***, ***H***) data for a representative subject. Power-law scaling was also highly significant for both the approach and avoidance data when ***H*** was set as the independent variable in place of ***K*** (Supplementary Table S2; all *p* < 10^−21^ for exponential term). These models indicate there is a significant inter-relationship between ***K*** and ***H***.

### Relationships among reward/aversion and divided attention measures

#### Iterative modeling of K, H, and β measures

To compare subjects' reward/aversion behavior to their performance on the divided attention task, we first looked for relationships between the reward/aversion measures *K* and *H* and the response bias, β, from the divided attention task. To understand the role of β in influencing subjects' relative preference behavior, we initially assessed whether there were significant one-way associations between β and K or H separately. Here, we used a power-law model of the form ***Y*** = ***a X***^*b*^ (where Y and X are the dependent and independent variables, respectively) to allow for the possibility of a nonlinear relation between variables. We tested these models using β as both the dependent and independent variable, and fit separate models for the approach (***K***^+^, ***H***^+^) and avoidance (***K***^−^, ***H***^−^) relative preference data. First, we tested whether there was any significant power-law scaling between β and H, and found no significant relationships for either the approach or avoidance data whether β or H was assigned as the independent variable (Supplementary Tables S3, S4; all *p* > 0.46 for exponential term). Second, we tested whether any significant relations existed between β and K^+^ or between β and K^−^. In this case, we found no significant relationships between β and K^+^ whether β was defined as the dependent variable or independent variable (Supplementary Tables S5, S6; *p* > 0.498). However, we did find significant power-law scaling between β and K^−^ whether defining β as either the dependent or independent variable (Supplementary Tables S5, S6; *p* = 0.0265). This relationship was robust to multiple comparisons corrections (Methods) accounting for all the statistical analyses performed, with a *q*-value of 0.0304 (Methods; Supplementary Tables S5, S6).

Given that none of the one-way relations between β and the relative preference measures (***K*** and ***H***) were consistently significant across both approach (***K***^+^, ***H***^+^) and avoidance (***K***^−^, ***H***^−^) data, we decided to focus on models that included both K and β as explanatory (i.e., independent) variables in order to evaluate how they together influence H. Indeed, we found that for both approach and avoidance data, the coefficients of β and K are consistently significant in these models (Table 2), which we describe below.

**Figure 3:**
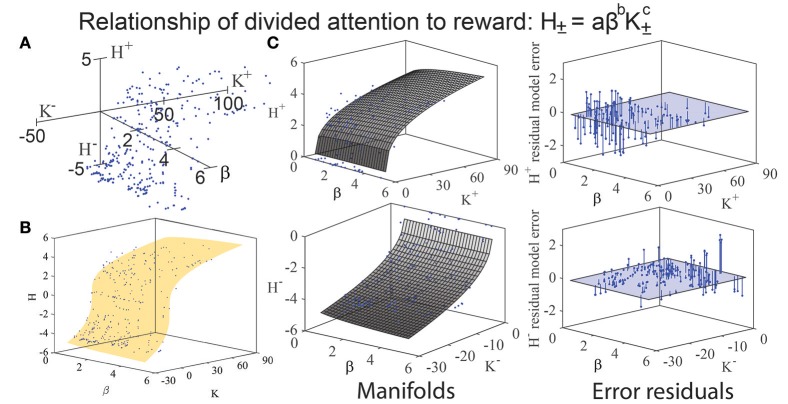
**(A)** When K and H (from Figures 1B, 2) are plotted against β on a third (z) axis, a manifold emerges. **(B)** Fitting of a multiplicative power-law model to the (***K, H*, β**) data of the form H = aβ^b^K^c^ reveals curvature along a manifold in three-dimensional space, reflecting an association between H and b. **(C)** The twisting of the observed manifolds is made more distinct by plotting the error residuals of ***H***^±^ from the multiplicative power-law model (H = aβ^b^K^c^) for approach (top right) and avoidance (bottom right). The approach manifold resembles a Cobb-Douglas graph. Note that the error residuals for ***H***^±^ are largest for small values of ***K*** (i.e., face categories of low mean keypressing), and become smaller at high values of ***K***. This indicates that the multiplicative power-law model can most reliably predict entropy (***H***) when keypressing is high in intensity (i.e., large magnitude of ***K***), and becomes less accurate as mean keypress intensity decreases.

We assessed the relationship of K, H, and β through iterative modeling (Banks and Tran, 2009), as done on prior occasions to determine if any mathematical structure existed such as a manifold, function, or boundary envelope (Kim et al., 2010). Given the observation of apparent manifolds for the positive (approach) and negative (avoidance) components of this function (Figures 3A,B), we attempted to characterize the observed relationship between these variables by fitting three mathematical functions to the data: (a) a logarithmic relationship (H = log a + b·log β + c·log K), (b) a multiplicative power law relationship (H = a·β^b^·K^c^; Figures 3B–C), and (c) an additive power law relationship (H = a +β^b^ + K^c^). The variables *a, b*, and *c* in these equations indicate fitted model coefficients. Fits and goodness of fit metrics for these three models are tabulated in Tables 2, 3.

**Table 3:** **Model fits for H^+^ and H^−^ using different functional forms**.

**Dependent Variable**	**Functional Form**	**RMSE**	**R**
H^+^	Logarithmic	0.8682	0.8079
H^−^	Logarithmic	0.5108	0.9112
H^+^	Power multiplicative	0.8667	0.8086
H^−^	Power multiplicative	0.5656	0.8899
H^+^	Power additive	0.8675	0.8082
H^−^	Power additive	0.5930	0.8782

*Root Mean Square Error (RMSE) and R are both measures of model fit. R is the Coefficient of Determination and is the square root of the ratio of Regression Sum of Squares (SSR) to Total Sum of Squares (SST)*.

Across the three models, we consistently found that K and β as explanatory variables explained 65–83% of the variance in H. To verify the stability of the model fits, we repeated the model fitting using an iterative least squares procedure that utilized random number generation to produce initial parameter estimates; repeated iterations of this randomized procedure produced consistent results with no changes in the estimated model coefficients.

All three of the models we tested had high F values (127–421 for approach data and 300–2250 for avoidance; see Table 2), with the multiplicative power-law model having the highest F value. On the other hand, all three models showed similar goodness of fit values (see RMSE values in Table 3). RMSE values were especially similar between the models when fitting the approach (***K***^+^, ***H***^+^) data: 0.8682 for logarithmic, 0.8667 for multiplicative power-law, and 0.8675 for additive power-law models. Given the similarity observed in goodness of fits, we did not perform formal model comparisons in an attempt to identify the best model for this dataset. In order to definitively compare these models, more statistical power would be required by repeating the experiment on a much larger cohort of subjects.

While we were unable to compare the model fits formally, we selected the multiplicative power-law model (i.e., H_±_ = a·β^b^·${\text{K}}_{\pm }^{\text{c}}$) for further study due to theoretical reasons. This multiplicative power-law function is commonly referred to in economics as the Cobb-Douglas production function (Cobb and Douglas, 1928). In particular, the exponential terms *b* and *c* of the Cobb-Douglas production function can be interpreted in terms of *resource matching*: when *b* + *c* < 1, the production function has what is called *inelasticity* (i.e., decreasing returns to scale), meaning that large changes to either of the dependent variables (i.e., β or *K*) will have comparatively only small effects on the output variable (i.e., *H*). Among the three models tested, this model exhibited the lowest rank root mean square error (RMSE) for the approach data, and the second lowest rank RMSE for the avoidance data. The F-statistic for the multiplicative power-law models for the approach data and avoidance data were also multiples of the other models (Table 2). However, it is worth re-emphasizing that our observed RMSE values were quite similar across the three models considered and thus no formal model comparison tests were performed; our choice of this model for further study was based purely on theoretical grounds.

Following our evaluation of model goodness of fits and selection of the multiplicative power-law model for further study, we used the parameter fits obtained from fitting the multiplicative power-law to get a better understanding of how β influences *H*^+^, *H*^−^, and by extension, proxy measures of a metric we refer to as the predictability bias (Methods). In particular, we plugged in the fitted model constants as well as the average observed value of *K*^+^ into the multiplicative power-law equation in order to predict *H*^+^, *H*^−^ (Figure 3A) the ratio proxy metric (*H*^−^/*H*^+^), and the difference proxy metric (*H*^−^–*H*^+^; Figure 3B). This procedure is described fully in the Methods. Robustness checks, which included using the mean value of *K*^−^ rather than that of *K*^+^, and using the intercept estimated from the avoidance model rather than that of the approach model, did little to change the results (not shown).

#### Relationship of H^−^, H^+^, and β

**Figure 4:**
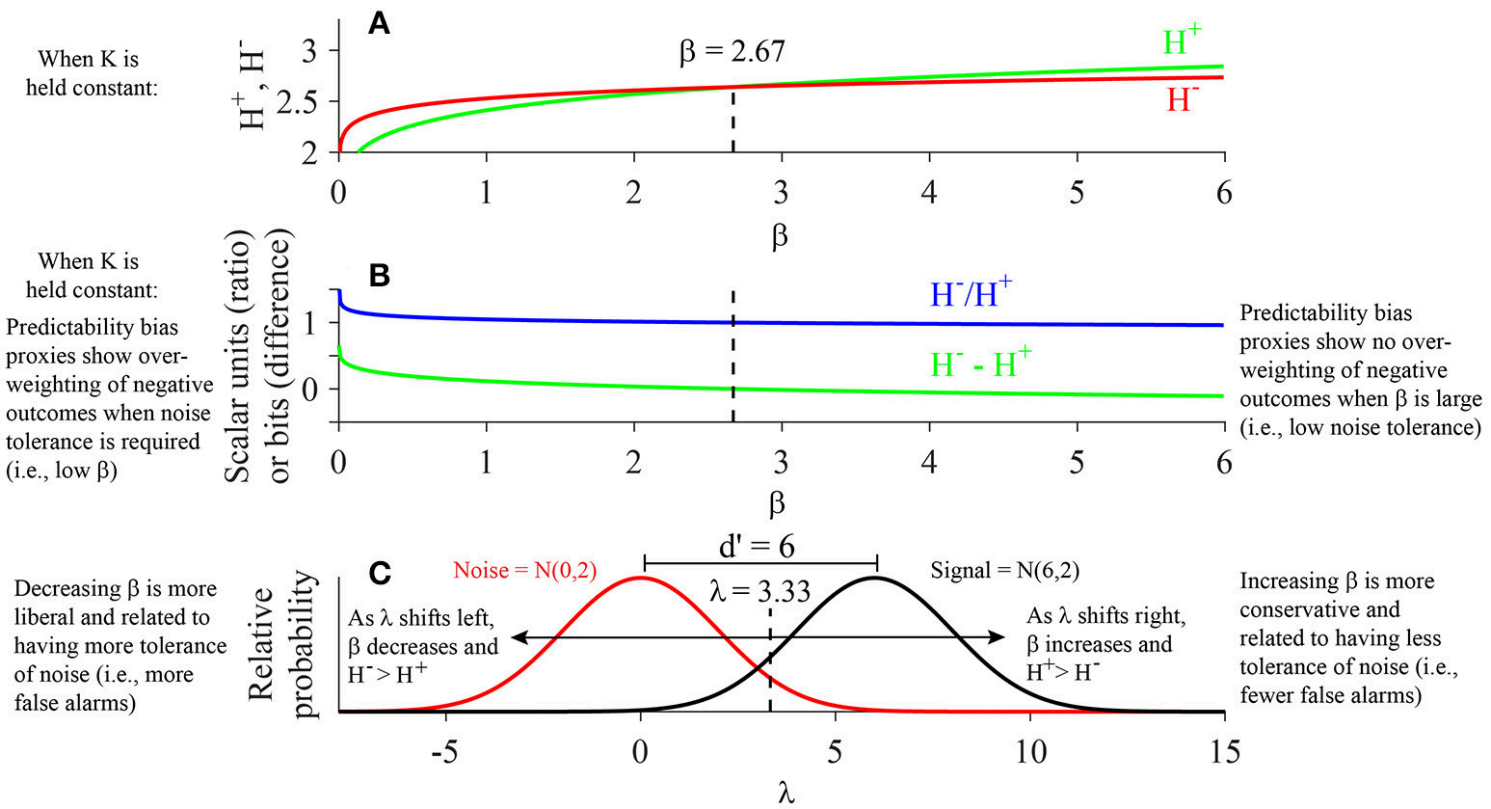
**Relationship of K, H, and β: Evaluation of the Cobb-Douglas Function. (A)** After inserting the respective estimated coefficients for the Cobb-Douglas model (H = aβ^b^K^c^) and plugging in the mean value of ***K***^+^ observed across all subjects and face categories, the approach (***H***^+^) and avoidance (***H***^−^) entropies are observed to intersect when β = 2.67. ***H***^−^exceeds ***H***^+^ for β < 2.67, while ***H***^+^exceeds ***H***^−^ for β > 2.67. **(B)** Plot displays ratio (***H***^−^/***H***^+^) and difference (***H***^−^**–*H***^+^) proxies for predictability bias as a function of β. The model predicts that the average subject exhibits high predictability bias (i.e., ***H***^−^***/H***^+^ > 1 or ***H***^−^**–*H***^+^ > 0) when β < 2.67, as opposed to low predictability bias (i.e., ***H***^−^***/H***^+^ < 1 or ***H***^−^**–*H***^+^ < 0) when β > 2.67. **(C)** An example signal detection schema is shown for a noise distribution of N(0,2) and signal distribution of N(6,2). Given equal variance in the signal and noise distributions, the d' is equal to the difference between the means of the signal and noise distributions (i.e., d′ = 6). The decision threshold corresponding to the critical β value of 2.67 is equal to 3.33 in this scenario (Results).

When isolating the effects of β on *H*^−^and *H*^+^ using our predictive model (Figure 4A), one observes that while the values of *H*^−^ are higher than *H*^+^ for small values of β, *H*^+^ values increase at a faster rate than *H*^−^ as β increases. One can look at the ratio *H*^−^/*H*^+^ and the difference *H*^−^ *- H*^+^ as proxies for the measure of predictability bias (Methods), which is analogous to the metric of *loss aversion* defined under prospect theory (Methods; Kahneman and Tversky, 1979; Tversky and Kahneman, 1991). Under our model, ***H***^−^ and ***H***^+^ are predicted to be of equal magnitude when β equals 2.67 (Figure 4A). At this critical value of β, the predicted ratio ***H***^−^/***H***^+^ equals 1 and their difference (***H***^−^–***H***^+^) equals 0 bits (Figure 4B). In other words, when one holds ***K***^+^ and ***K***^−^ constant and evaluates the relationship of β to ***H***^+^ and ***H***^−^, one observes that for β less than 2.67 (i.e., a more liberal response bias producing higher hit and false alarm rates), ***H***^−^ is greater than ***H***^+^, indicating a predictability bias on the approach/avoidance task in which avoidance keypressing is more predictable than approach keypressing, analogous to an increase in loss aversion (Tversky and Kahneman, 1991, 1992). In contrast, when β exceeds 2.67 (i.e., a more conservative response bias resulting in lower hit and false alarm rates), ***H***^+^ is greater than ***H***^−^, suggesting approach behavior becomes more predictable relative to avoidance behavior, analogous to a decrease in loss aversion.

To complete our modeling analysis of *K, H*, and beta variables, we used the values of β observed in Figures 4A,B to estimate the decision threshold λ. Specifically, we estimated the decision threshold λ for a scenario where the distribution of noise is normally distributed with a mean of 0 and standard deviation of 2, and the signal distribution is normally distributed with a mean of 6 and standard deviation of 2 (Figure 4C). First, we can calculate the parameter *d*′ as the distance between the means of the two distributions: d′ = 6–0 = 6. β is calculated as $\beta =\frac{f_{s}\left(\lambda \right)}{f_{n}\left(\lambda \right)}$, where *f*_*s*_(λ) and *f*_*n*_(λ) are the probability mass functions for the signal and noise distributions, respectively. Thus, β is defined as the ratio of the heights of the two distributions at the threshold criterion λ. Note that we have already estimated the critical β value directly from our model fits as β = 2.67. Given this value, we can then compute the decision threshold λ corresponding to the critical β value by using the following formula:

(7) $$\lambda =\frac{\sigma \;\ln(\beta )}{d\prime }\;+\;\frac{\mu_{s}+\mu_{n}}{2},$$

where σ is the standard deviation of the signal and noise distributions (which are equal in this scenario), and μ_*s*_ and μ_*n*_ are the means of the signal and noise distributions, respectively. Plugging in the observed values for these parameters under the scenario depicted in Figure 4C as well as the critical β value of 2.67 obtained from our empirical model into Equation 7, we obtain a decision threshold of λ = 3.33.

Importantly, this decision threshold (λ = 3.33) is closer to the signal distribution than the noise distribution, indicating that under our model, a subject with a neutral predictability bias (***H***^−^***/H***^+^ = 1 or ***H***^−^–***H***^+^ = 0) is expected to tend toward a more conservative response bias (i.e., will achieve fewer false alarms and more correct rejections at the cost of more misses and fewer hits of the true signal). Conversely, the model results displayed in Figure 4 suggest that a subject with a neutral decision threshold (i.e., a threshold equidistant from the noise and signal distributions; in this case, λ = 3) would be expected to exhibit predictability bias greater than one. This is true because a neutral decision threshold in this case would require λ to decrease from 3.33 to 3, resulting in a corresponding decrease in β (i.e., β = 1 for a neutral decision threshold). As shown in Figure 4, a decrease in λ and β coincides with an increase in ***H***^−^ relative to ***H***^+^ (Figure 4A), and hence an increase in predictability bias (Figure 4B).

#### Relationship of exponents under multiplicative power law model

It is also informative to consider the magnitudes of the exponential terms *b* and *c* in our fitted power-law model. Under the multiplicative power law model we fit for theoretical reasons (i.e., *H* = *a*β^*b*^*K*^*c*^), we observed that the exponential model coefficients *b* and *c* summed to less than one for both the approach (b + c = 0.092 + 0.319 = 0.411) and avoidance (b + c = 0.044 + 0.337 = 0.381) models. In Cobb-Douglas resource matching terms (Cobb and Douglas, 1928), the fact that the sums of these exponential coefficients are less than 1 indicates that overall, individuals show decreasing returns to scale or “inelasticity,” resulting in attenuated relative increases of ***H***^±^ despite substantial increases in ***K***^±^ or β.

#### Iterative modeling of K, H, and d′ measures

To ensure there were no other significant relationships between K and H variables from the approach/avoidance task and other parameters estimated from the divided attention task, we also assessed for one- and two-way relations between d′ (sensitivity) from the divided attention task and K and H from the approach/avoidance task. We observed no significant relationships between these variables (Supplementary Tables S7–S11). When assessing power-law relationships between d′ and H, the exponential terms were not significantly different from zero for either the approach or avoidance data whether d' was assigned as the dependent or independent variable (Supplementary Tables S7, S8; all *p* > 0.32). Likewise, the exponential terms did not differ significantly from zero for any power-law models relating d' and K (Supplementary Tables S9, S10; all *p* > 0.119). After assessing for potential one-way relations between d′ and the relative preference measures, we next considered two-dimensional relationships by fitting models that related H, K, and d′ (i.e., models of the form H = f(K, d′)). Specifically, we fit the same three models that we considered previously when evaluating the relationship between K, H, and β: (a) a logarithmic relationship (H = log a + b·log d′ + c·log K), (b) a multiplicative power law relationship (H = ad′^b^K^c^), and (c) an additive power law relationship (H = a + d′^b^ + K^c^). Notably, the model coefficients (*b*) representing the d′ variable were not significantly different from 0 under any model for either the approach or avoidance data (Supplementary Table S11; all *p* > 0.28), indicating that d′ had no influence on predicting ***H***^±^ even in the context of a two-dimensional model with ***K***^±^.

#### Multiple comparisons corrections

All 20 of the univariate and multivariate *p*-values relating divided attention and reward/aversion metrics went into our FDR analysis to account for all of the statistical tests we performed. The resulting *q*-values are listed below their corresponding raw *p*-values in Table 2 and Tables S1–S11. Of the 20 statistical tests we performed, only the multivariate models of the form H = f(K, β) were putatively significant based on raw *p*-values. The univariate models linking these variables as well as the multivariate models for H = f(K, d′) were not even remotely close to achieving significance for the relevant parameters (with the exception of power-law scaling between ***K***^−^ and β, discussed earlier), and we therefore limit our discussion here to the q-values observed for the H = f(K, β) models. As shown in Table 2, all ***b*** coefficients (exponential term for the β parameter) had associated *q*-values of less than 0.05 for all three types of H = f(K, β) models (whether considering the approach or avoidance keypress data), with the exception of the multiplicative power-law model for the approach data, which had a q-value of 0.0557. Thus, the observed significance levels of the β parameters in these three models can be accepted with confidence that less than 5% of these observations are expected to be false positives (with the exception of the multiplicative power-law model fit to approach data, for which this confidence level drops to 5.57%).

## Discussion

In this paper, we explored the relationship between measures of reward/aversion behavior from an approach/avoidance keypress task (i.e., ***K*** and ***H***) and measures of signal detection performance from a divided attention task (i.e., false alarm rate, β, and d′). We found no significant relations between d′ under signal detection and reward/aversion variables, but through iterative modeling, we identified significant relationships between K, H, and β, in particular of β on H, in parallel with K on H. We considered in particular a multiplicative power-law model (H = aβ^b^K^c^) known in economics as the Cobb-Douglas production function. Plugging in our estimated model coefficients and the mean observed value of ***K*** allowed us to predict ***H***^±^ as a function of β. Doing so revealed that the response bias (β) exerts an effect on the relative magnitudes of approach (***H***^+^) and avoidance (***H***^−^) entropies, which in turn can be used as proxy measures to describe how predictable or consistent subjects' avoidance behaviors are relative to their approach behaviors on the keypress task (i.e., predictability bias). Namely, lower values of β were predictive of a greater predictability bias (analogous to greater loss aversion) on the approach/avoidance task, higher values of β predicted lower levels of predictability bias, and a neutral predictability bias (i.e., ***H***^−^ = ***H***^+^) was expected at a critical β value of 2.67.

It is worth noting that our effort is not the first to draw connections between attention or signal detection and reward/aversion processing. A particularly interesting study was conducted by Navalpakkam et al. (2009) that examined how subjects perform on a signal detection task related to visual attention in the context of variable reward schemes. The authors demonstrated that subjects' target detection rates could be specifically modulated by adjusting the reward/penalty policies associated with the task, and that human participants quickly arrived at the statistically optimal decision strategy (Navalpakkam et al., 2009). This study thus established a clear, quantitative, and directed link between reward and performance on a visual attention task. Moreover, foundational work by Busemeyer and Townsend as well as teams led by Usher, McClelland, Rangel and others have also included frameworks emphasizing attentional models of reward and value-based choice (Busemeyer and Townsend, 1993; Roe et al., 2001; Usher and McClelland, 2001, 2004; Krajbich et al., 2010, 2012; Milosavljevic et al., 2010; Krajbich and Rangel, 2011; Vincent, 2015). Such work has involved both linear (e.g., Krajbich et al., 2010, 2012; Krajbich and Rangel, 2011) and non-linear (e.g., Busemeyer and Townsend, 1993; Usher and McClelland, 2001, 2004) diffusion models of evidence accumulation bearing on value-based decisions (see Vincent, 2015 for recent review). In particular, Usher and McClelland have investigated the role of loss aversion (analogous to predictability bias) in multi-alternative value-based choice under a diffusion framework (Usher and McClelland, 2004).

These and related models offer statistical accounts for how attention or attention switching may lead to accumulation of evidence favoring particular alternatives in value-based choice, and make powerful predictions of choice behavior at the single-trial level. However, it is important to note fundamental differences in scope between these existing frameworks and our application of RPT (Kim et al., 2010) in the present study. While the aforementioned diffusion models offer dynamic, statistical frameworks providing post-hoc explanations for value-based decision-making, our aim in using a relative preference (i.e., approach/avoidance) task was rather to characterize the broad emotional profile of individual preferences toward a range of objects, within a model that allows inference and is lawful (Kim et al., 2010), and is completely derived from empirical data with a keypress task, an approach that has been considered a gold-standard for neuroscience study of reward/aversion (e.g., White et al., 1987). The keypress task we used was developed within a behaviorist operant framework (Aharon et al., 2001; Lee et al., 2015). With the RPT analysis, we utilized an information theory variable (Shannon entropy) to characterize individuals' patterns of approach and avoidance behavior across multiple categories of face stimuli. We view diffusion models of evidence accumulation toward a threshold of value-based choice as modeling an important aspect of motivated behavior, one that might be strengthened if integrated with the multidimensional modeling of preference and its control functions represented by RPT. Integration of diffusion evidence accumulation with RPT valuation may be of potential interest for future studies.

In considering our findings in the context of existing work, it is important to acknowledge that additional or intermediate variables may exist which moderate or mediate relations between attention deployment and reward functions. This may facilitate study of directional effects between these variables. We did not find a relationship between **K**, **H** and **d**′, but it is possible other variables in RPT may have such a relationship. For instance, the **K** and **σ** variables in RPT form a variance-mean graph that appear to encode features of Markowitz's decision utility (Kim et al., 2010), and might be hypothesized to have a relationship with **d**′ which represents the separation of signal and noise distributions. Future work is clearly needed to assess this, including work done outside of large-scale phenotype genotype projects that counterbalance many experiments beyond those discussed here. In our study, all stimulus presentation in the MRI was counterbalanced across subjects, but the possibility exists that completing tasks in a supine position may influence the behavior performance we report.

Although our study investigated attention and reward at the behavioral level, it is interesting to speculate on the neural pathways and mechanisms that may be involved in the relationships we observed between these two domains. The existing literature strongly suggests frontal and parietal areas (notably, the intraparietal sulcus and ventromedial prefrontal cortex) as being preferentially activated in high-reward conditions during attentional states (Taylor et al., 2004; Small et al., 2005; Engelmann et al., 2009), or conversely as being modulated by the difference in perceived reward by the ventral striatum between attended and unattended items in a choice task (Lim et al., 2011). Although we can only speculate, it is possible that underlying physiological differences in pathways involving these brain regions exert common effects that lead to systematic differences in both reward and attention behaviors across individuals. The finding of β affecting H in our study, shows an effect of attention on reward, which complements the strong data showing reward affecting attention (Taylor et al., 2004; Small et al., 2005; Engelmann and Pessoa, 2007; Engelmann et al., 2009). These brain regions also serve as a subset of those implicated in emotion more generally (e.g., Breiter and Rosen, 1999; Ochsner and Gross, 2005; Oosterwijk et al., 2012), raising interesting parallels between modern hypotheses about emotion as a relationship between systems for reward/aversion, memory, and attention (Breiter and Rosen, 1999; Russell and Barrett, 1999; Breiter et al., 2006; Barrett et al., 2007; Gross and Barrett, 2011) and the current model between {***K***^±^, ***H***^±^, β} variables observed here.

Two general implications arise from the present findings. The first relates to modeling the relations of variables in the same manner as is done in mechanistic disciplines (e.g., pressure, temperature, and volume with the gas laws in chemistry). When we do this, predictability bias in the framework of relative preference theory (which resembles loss aversion in prospect theory Kahneman and Tversky, 1979; Tversky and Kahneman, 1991) appears to be associated with how individuals set their response bias during the divided attention task. The more β decreases, consistent with an increasing tolerance for noise during divided attention, the more avoidance entropy (***H***−) increases relative to approach entropy (**H**+) on the approach/avoidance task, which in the context of equal keypressing intensity (i.e., ***K***^+^ = ***K***^−^ = constant), implies that the slope of the avoidance curve is steeper than the slope of the approach curve on the (***K***, ***H***) value function. These data argue that subjects who exhibit greater predictability bias during the approach/avoidance task may also tend to have a more liberal response bias during the divided attention task (i.e., smaller β). When individuals are more conservative on the divided attention task and the false alarm rate is low (i.e., large β), *H*^+^ is prioritized relative to *H*^−^, and these individuals' patterns of approach behavior become relatively more predictable when compared to their avoidance behaviors on the keypress task (e.g., analogous to having less loss aversion). Altogether, our study suggests that the decision threshold (demarcating the trade-off between hit and false alarm rates) that is chosen by subjects during divided attention may be closely linked to the predictability bias observed in the same individuals during reward/aversion behavior.

A second implication arises when one considers that the multiplicative power-law relationship evaluated in our study (i.e., H = aβ^b^K^c^) has the same format as the Cobb-Douglas production function in economics (Cobb and Douglas, 1928). In economics, the Cobb-Douglas function has been broadly applied in applications ranging from matching theory for describing mutually beneficial relationships (Mortensen and Pissarides, 1994) to modeling the private domestic sector of the US economy from 1929 to 1967 (Sinai and Stokes, 1972). An important feature of the Cobb-Douglas function is that the power law exponents together determine the relationship between the input variables (i.e., *K*^±^ and β) and the output variable (*H*^±^) as a type of resource matching operation (Cobb and Douglas, 1928). For instance, the output variable increases proportionally with the input variables (i.e., the output variable doubles when the input variables double) if the exponents b + c = 1. If b + c < 1, the output variable will show decreasing returns to scale or “inelasticity”: in this case, there will be relatively small changes in *H*^±^ despite substantial changes in *K*^±^ and β. For our data, b + c << 1 for both approach and avoidance data, raising the hypothesis of a control function defining capacity constraints (Mortensen and Pissarides, 1994) to processing for divided attention and reward/aversion behavior, and potentially suggesting a mechanism for the capacity constraints to attention hypothesized by Kahneman (1973).

In conclusion, our data suggest that a systematic relationship exists between quantitative formulations of reward/aversion behavior and divided attention as gauged by independent approach/avoidance and divided attention tasks carried out in the same cohort of subjects. The relationship uncovered in the present study underscores why concerns have arisen about the potential interplay of attention with reward variables in psychology and neuroscience (Maunsell, 2004), and supports the need for further study. It raises at least two issues; first, when reward or attention tasks are performed in isolation, the results may bear the caveat of the other function not being controlled in the experiment. Second, the relationships we report between divided attention and relative preference variables in Figures 3, 4 were accomplished through exploratory, iterative modeling of various possible associations among these variables. Our success using this approach builds upon existing efforts that have identified relationships across behavioral domains, and further demonstrates how quantitative approaches permit complex associations between variables spanning many domains of human behavior to be rigorously assessed and identified.

## Author contributions

Developed analytical methods and concepts for experimental design and analysis in manuscript: VV, JS, BK, CP, HY, ML, KR, FM, MB, BC, SL, DM, AB, and HB. Developed analytical methods and computer code for data collection: BK, ML. Analyzed data: VV, JS, BK, CP, ML, and HB. Wrote manuscript: VV, JS, CP, AB, and HB.

### Conflict of interest statement

The authors declare that the research was conducted in the absence of any commercial or financial relationships that could be construed as a potential conflict of interest.

## References

[B1] Abdellaoui M. Bleichrodt H. Paraschiv C. (2007). Loss aversion under prospect theory: a parameter-free measurement. Manage. Sci. 53, 1659–1674. 10.1287/mnsc.1070.0711

[B2] Aharon I. Etcoff N. Ariely D. Chabris C. F. O'Connor E. Breiter H. C. (2001). Beautiful faces have variable reward value: fMRI and behavioral evidence. Neuron 32, 537–551. 10.1016/S0896-6273(01)00491-3 11709163

[B3] Bandura A. (1997). Self-efficacy: The Exercise of Control. New York, NY: Freeman.

[B4] Banks H. T. Tran H. T. (2009). Mathematical and Experimental Modeling of Physical and Biological Processes. Boca Raton, FL: CRC Press.

[B5] Barrett L. F. Mesquita B. Ochsner K. N. Gross J. J. (2007). The experience of emotion. Annu. Rev. Psychol. 58, 373–403. 10.1146/annurev.psych.58.110405.085709 17002554 PMC1934613

[B6] Beck L. H. Bransome E. D. Jr. Mirsky A. F. Rosvold H. E. Sarason I. (1956). A continuous performance test of brain damage. J. Consult. Psychol. 20, 343–350. 10.1037/h0043220 13367264

[B7] Benartzi S. Thaler R. H. (1995). Myopic loss aversion and the equity premium puzzle. Q. J. Econ. 110, 73–92. 10.2307/2118511

[B8] Benjamini Y. Hochberg Y. (1995). Controlling the false discovery rate: a practical and powerful approach to multiple testing. J. R. Statist. Soc. Ser. B (Methodol.) 57, 289–300. 10.2307/2346101

[B9] Benson V. Marano M. A. (1998). Current estimates from the National Health Interview Survey. Vital Health Stat. 10, 1–428. 9914773

[B10] Biederman J. (1992). Further evidence for family-genetic risk factors in attention deficit hyperactivity disorder. Arch. Gen. Psychiatry 49, 728. 10.1001/archpsyc.1992.01820090056010 1514878

[B11] Booij A. S. van de Kuilen G. (2009). A parameter-free analysis of the utility of money for the general population under prospect theory. J. Econ. Psychol. 30, 651–666. 10.1016/j.joep.2009.05.004

[B12] Breiter H. C. (2012). Reward/Aversion Processing during Decision-Making: A New Framework and Its Biological Implications. Boston, MA: Presentation at Boston University.

[B13] Breiter H. C. (2014). Developing a quantitative model of the mind and its implications for neuroscience, in Plenary Talk for 2014 Meeting of the Chicago Society for Neuroscience (Chicago, IL).

[B14] Breiter H. C. Gasic G. P. Makris N. (2006). Imaging the neural systems for motivated behavior and their dysfunction in neuropsychiatric illness, in Complex Systems Science in Biomedicine, eds Deisboeck T. S. Kresh J. Y. (Boston, MA: Kluwer Academic Publishers), 763–810.

[B15] Breiter H. C. Rosen B. R. (1999). Functional magnetic resonance imaging of brain reward circuitry in the human. Ann. N.Y. Acad. Sci. 877, 523–547. 10.1111/j.1749-6632.1999.tb09287.x 10415669

[B16] Busemeyer J. R. Townsend J. T. (1993). Decision field theory: a dynamic-cognitive approach to decision making in an uncertain environment. Psychol. Rev. 100, 432–459. 10.1037/0033-295X.100.3.432 8356185

[B17] Christensen A. Giese M. A. Sultan F. Mueller O. M. Goericke S. L. Ilg W.. (2014). An intact action-perception coupling depends on the integrity of the cerebellum. J. Neurosci. 34, 6707–6716. 10.1523/JNEUROSCI.3276-13.2014 24806697 PMC6608134

[B18] Cobb C. W. Douglas P. H. (1928). A theory of production. Am. Econ. Rev. 18, 139–165.

[B19] Cohen J. D. Barch D. M. Carter C. Servan-Schreiber D. (1999). Context-processing deficits in schizophrenia: converging evidence from three theoretically motivated cognitive tasks. J. Abnorm. Psychol. 108, 120–133. 10.1037/0021-843X.108.1.120 10066998

[B20] Cook E. H. Jr. Stein M. A. Krasowski M. D. Cox N. J. Olkon D. M. Kieffer J. E.. (1995). Association of attention-deficit disorder and the dopamine transporter gene. Am. J. Hum. Genet. 56, 993–998. 7717410 PMC1801209

[B21] Croxson P. Walton M. O-Reilly J. Behrens E. Rushworth M. (2009). Effort-based cost-benefit valuation and the human brain. J. Neurosci. 29, 4531–4541. 10.1523/JNEUROSCI.4515-08.2009 19357278 PMC2954048

[B22] Davies D. R. Parasuraman R. (1982). The Psychology of Vigilance. London; New York, NY: Academic Press.

[B23] Deci E. L. Ryan R. M. (1985). Intrinsic Motivation and Self-Determination in Human Behavior. New York, NY: Plenum Press.

[B24] Eckstein M. P. Thomas J. P. Palmer J. Shimozaki S. S. (2000). A signal detection model predicts the effects of set size on visual search accuracy for feature, conjunction, triple conjunction, and disjunction displays. Percept. Psychophys. 62, 425–451. 10.3758/BF03212096 10909235

[B25] Elman I. Ariely D. Mazar N. Aharon I. Lasko N. B. Macklin M. L.. (2005). Probing reward function in post-traumatic stress disorder with beautiful facial images. Psychiatry Res. 135, 179–183. 10.1016/j.psychres.2005.04.002 15993948

[B26] Engelmann J. B. Damaraju E. Padmala S. Pessoa L. (2009). Combined effects of attention and motivation on visual task performance: transient and sustained motivational effects. Front. Hum. Neurosci. 3:4. 10.3389/neuro.09.004.2009 19434242 PMC2679199

[B27] Engelmann J. B. Pessoa L. (2007). Motivation sharpens exogenous spatial attention. Emotion 7, 668–674. 10.1037/1528-3542.7.3.668 17683222

[B28] First M. B. Spitzer R. Gibbon M. (1996). Structured clinical interview for DSM-IV Axis I Disorders, Research Version, Non-patient Edn. (SCID-I/NP) in B. R. Department. New York, NY: New York State Psychiatric Institute.

[B29] Gasic G. P. Smoller J. W. Perlis R. H. Sun M. Lee S. Kim B. W.. (2009). BDNF, relative preference, and reward circuitry responses to emotional communication. Am. J. Med. Genet. B Neuropsychiatr. Genet. 150B, 762–781. 10.1002/ajmg.b.30944 19388013 PMC7891456

[B30] Gossop M. Marsden J. Stewart D. Kidd T. (2003). The National Treatment Outcome Research Study (NTORS): 4-5 year follow-up results. Addiction 98, 291–303. 10.1046/j.1360-0443.2003.00296.x 12603229

[B31] Green D. Swets J. (1966). Signal Detection and Psychophysics. New York, NY: Wiley.

[B32] Gross J. J. Barrett L. F. (2011). Emotion generation and emotion regulation: one or two depends on your point of view. Emot. Rev. 3, 8–16. 10.1177/1754073910380974 21479078 PMC3072688

[B33] Halperin J. M. Wolf L. E. Pascualvaca D. M. Newcorn J. H. Healey J. M. O'Brien J. D.. (1988). Differential assessment of attention and impulsivity in children. J. Am. Acad. Child Adolesc. Psychiatry 27, 326–329. 10.1097/00004583-198805000-00010 3379014

[B34] Kahneman D. (1973). Attention and Effort. Englewood Cliffs, NJ: Prentice-Hall.

[B35] Kahneman D. Tversky A. (1979). Prospect theory: an analysis of decision under risk. Econometrica 47, 263 10.2307/1914185

[B36] Kawagoe R. Takikawa Y. Hikosaka O. (1998). Expectation of reward modulates cognitive signals in the basal ganglia. Nat. Neurosci. 1, 411–416. 10.1038/1625 10196532

[B37] Kim B. W. Kennedy D. N. Lehár J. Lee M. J. Blood A. J. Lee S.. (2010). Recurrent, robust and scalable patterns underlie human approach and avoidance. PLoS ONE 5:e10613. 10.1371/journal.pone.0010613 20532247 PMC2879576

[B38] Köbberling V. Wakker P. P. (2005). An index of loss aversion. J. Econ. Theory 122, 119–131. 10.1016/j.jet.2004.03.009

[B39] Krajbich I. Armel C. Rangel A. (2010). Visual fixations and the computation and comparison of value in simple choice. Nat. Neurosci. 13, 1292–1298. 10.1038/nn.2635 20835253

[B40] Krajbich I. Lu D. Camerer C. Rangel A. (2012). The attentional drift-diffusion model extends to simple purchasing decisions. Front. Psychol. 3:193. 10.3389/fpsyg.2012.00193 22707945 PMC3374478

[B41] Krajbich I. Rangel A. (2011). Multialternative drift-diffusion model predicts the relationship between visual fixations and choice in value-based decisions. Proc. Natl. Acad. Sci. U.S.A. 108, 13852–13857. 10.1073/pnas.1101328108 21808009 PMC3158210

[B42] Lee S. Lee M. J. Kim B. W. Gilman J. M. Kuster J. K. Blood A. J.. (2015). The commonality of loss aversion across procedures and stimuli. PLoS ONE 10:e0135216. 10.1371/journal.pone.0135216 26394306 PMC4579072

[B43] Levy B. Ariely D. Mazar N. Chi W. Lukas S. Elmana I. (2008). Gender differences in the motivational processing of facial beauty. Learn. Motiv. 39, 136–145. 10.1016/j.lmot.2007.09.002 24282336 PMC3838871

[B44] Lim S. L. O'Doherty J. P. Rangel A. (2011). The decision value computations in the vmPFC and striatum use a relative value code that is guided by visual attention. J. Neurosci. 31, 13214–13223. 10.1523/JNEUROSCI.1246-11.2011 21917804 PMC6623246

[B45] Makris N. Gasic G. P. Kennedy D. N. Hodge S. M. Kaiser J. R. Lee M. J.. (2008). Cortical thickness abnormalities in cocaine addiction—a reflection of both drug use and a pre-existing disposition to drug abuse? Neuron 60, 174–188. 10.1016/j.neuron.2008.08.011 18940597 PMC3772717

[B46] Maunsell J. H. (2004). Neuronal representations of cognitive state: reward or attention? Trends Cogn. Sci. (Regul. Ed). 8, 261–265. 10.1016/j.tics.2004.04.003 15165551

[B47] McNicol D. (1972). A Primer of Signal Detection Theory, Vol. 8. London: Allen and Unwin.

[B48] Milosavljevic M. Malmaud J. Huth A. Koch C. Rangel A. (2010). The drift diffusion model can account for the accuracy and reaction time of value-based choices under high and low time pressure. Judgment Decis. Making 5, 437–449. 10.2139/ssrn.1901533

[B49] Mortensen D. T. Pissarides C. A. (1994). Job creation and job destruction in the theory of unemployment. Rev. Econ. Stud. 61, 397–415. 10.2307/2297896

[B50] Navalpakkam V. Koch C. Perona P. (2009). Homo economicus in visual search. J. Vis. 9, 31–16. 10.1167/9.1.31 19271901

[B51] Ochsner K. N. Gross J. J. (2005). The cognitive control of emotion. Trends Cogn. Sci. 9, 242–249. 10.1016/j.tics.2005.03.010 15866151

[B52] Oldfield R. C. (1971). The assessment and analysis of handedness: the Edinburgh inventory. Neuropsychologia 9, 97–113. 10.1016/0028-3932(71)90067-4 5146491

[B53] Oosterwijk S. Lindquist K. A. Anderson E. Dautoff R. Moriguchi Y. Barrett L. F. (2012). States of mind: emotions, body feelings, and thoughts share distributed neural networks. NeuroImage 62, 2110–2128. 10.1016/j.neuroimage.2012.05.079 22677148 PMC3453527

[B54] Park Y. Waldman I. D. (2014). Influence of the COMT val108/158met polymorphism on continuous performance task indices. Neuropsychologia 61, 45–55. 10.1016/j.neuropsychologia.2014.06.008 24946318 PMC4122640

[B55] Perlis R. H. Holt D. J. Smoller J. W. Blood A. J. Lee S. Kim B. W.. (2008). Association of a polymorphism near CREB1 with differential aversion processing in the insula of healthy participants. Arch. Gen. Psychiatry 65, 882–892. 10.1001/archgenpsychiatry.2008.3 18678793 PMC3782742

[B56] Platt M. L. Glimcher P. W. (1999). Neural correlates of decision variables in parietal cortex. Nature 400, 233–238. 10.1038/22268 10421364

[B57] Ptak R. (2012). The frontoparietal attention network of the human brain: action, saliency, and a priority map of the environment. Neuroscientist 18, 502–515. 10.1177/1073858411409051 21636849

[B58] Raman K. (2013). Attention, reward and customer strategies for sustained growth [Oral presentation], in Customer Strategies for Sustained Growth (Fontainebleau: INSEAD).

[B59] Reeke G. N. Coop A. D. (2004). Estimating the temporal interval entropy of neuronal discharge. Neural Comput. 16, 941–970. 10.1162/089976604773135050 15070505

[B60] Rieke F. (1997). Spikes: Exploring the Neural Code, Vol. xvi Cambridge, MA: MIT Press.

[B61] Roe R. M. Busemeyer J. R. Townsend J. T. (2001). Multialternative decision field theory: a dynamic connectionist model of decision making. Psychol. Rev. 108, 370–392. 10.1037/0033-295X.108.2.370 11381834

[B62] Russell J. A. Barrett L. F. (1999). Core affect, prototypical emotional episodes, and other things called emotion: dissecting the elephant. J. Pers. Soc. Psychol. 76, 805–819. 10.1037/0022-3514.76.5.805 10353204

[B63] Schmidt U. Zank H. (2005). What is loss aversion? J. Risk Uncertain. 30, 157–167. 10.1007/s11166-005-6564-6

[B64] Seidman L. J. Breiter H. C. Goodman J. M. Goldstein J. M. Woodruff P. W. O'Craven K.. (1998). A functional magnetic resonance imaging study of auditory vigilance with low and high information processing demands. Neuropsychology 12, 505–518. 10.1037/0894-4105.12.4.505 9805320

[B65] Shannon C. E. Weaver W. (1949). The Mathematical Theory of Communication. Urbana, IL: University of Illinois Press.

[B66] Sinai A. Stokes H. H. (1972). Real money balances: an omitted variable from the production function? Rev. Econ. Stat. 54, 290.

[B67] Small D. M. Gitelman D. Simmons K. Bloise S. M. Parrish T. Mesulam M.-M. (2005). Monetary incentives enhance processing in brain regions mediating top-down control of attention. Cereb. Cortex 15, 1855–1865. 10.1093/cercor/bhi063 15746002

[B68] Smith G. D. Lambert J. V Moore Z. (2013). Behavior description effect on accuracy and reliability. J. Gen. Psychol. 140, 269–281. 10.1080/00221309.2013.818525 24837820

[B69] Storey J. D. (2002). A direct approach to false discovery rates. J. R. Statist. Society: Ser. B (Statist. Methodol.) 64, 479–498. 10.1111/1467-9868.00346

[B70] Strauss M. M. Makris N. Aharon I. Vangel M. G. Goodman J. Kennedy D. N.. (2005). fMRI of sensitization to angry faces. Neuroimage 26, 389–413. 10.1016/j.neuroimage.2005.01.053 15907298

[B71] Tanner W. P. Jr. Swets J. A. (1954). A decision-making theory of visual detection. Psychol. Rev. 61, 401–409. 10.1037/h0058700 13215690

[B72] Taylor S. F. Welsh R. C. Wager T. D. Phan K. L. Fitzgerald K. D. Gehring W. J. (2004). A functional neuroimaging study of motivation and executive function. Neuroimage 21, 1045–1054. 10.1016/j.neuroimage.2003.10.032 15006672

[B73] Tiesinga P. H. Fellous J. M. Jose J. V Sejnowski T. J. (2002). Information transfer in entrained cortical neurons. Network 13, 41–66. 10.1080/net.13.1.41.66 11878284

[B74] Tom S. M. Fox C. R. Trepel C. Poldrack R. A. (2007). The neural basis of loss aversion in decision-making under risk. Science 315, 515–518. 10.1126/science.1134239 17255512

[B75] Tversky A. Kahneman D. (1991). Loss aversion in riskless choice: a reference-dependent model. Q. J. Econ. 106, 1039–1061. 10.2307/2937956

[B76] Tversky A. Kahneman D. (1992). Advances in prospect theory: cumulative representation of uncertainty. J. Risk Uncertain. 5, 297–323. 10.1007/BF00122574

[B77] Usher M. McClelland J. L. (2001). The time course of perceptual choice: the leaky, competing accumulator model. Psychol. Rev. 108, 550–592. 10.1037/0033-295X.108.3.550 11488378

[B78] Usher M. McClelland J. L. (2004). Loss aversion and inhibition in dynamical models of multialternative choice. Psychol. Rev. 111, 757–769. 10.1037/0033-295X.111.3.757 15250782

[B79] Verghese P. (2001). Visual search and attention: a signal detection theory approach. Neuron 31, 523–535. 10.1016/S0896-6273(01)00392-0 11545712

[B80] Vincent B. T. (2015). Bayesian accounts of covert selective attention: a tutorial review. Attent. Percept. Psychophys. 77, 1013–1032. 10.3758/s13414-014-0830-0 25762302

[B81] Viola P. A. Schraudolph N. N. Sejnowski T. J. (1996). Empirical entropy manipulation for real-world problems, in Advances in Neural Information Processing Systems, eds Touretzkym D. S. Mozer M. Hasselmo M. (Cambridge, MA: The MIT Press), 851–857.

[B82] Viswanathan V. Lee S. Gilman J. M. Kim B. W. Lee N. Chamberlain L.. (2015). Age-related striatal BOLD changes without changes in behavioral loss aversion. Front. Hum. Neurosci. 9:176. 10.3389/fnhum.2015.00176 25983682 PMC4415398

[B83] Waelti P. Dickinson A. Schultz W. (2001). Dopamine responses comply with basic assumptions of formal learning theory. Nature 412, 43–48. 10.1038/35083500 11452299

[B84] Walton M. Kennerley S. Bannerman D. Phillips P. Rushworth M. (2006). Weighing up the benefits of work: behavioral and neural analyses of effort-related decision making. Neural Netw. 19, 1302–1314. 10.1016/j.neunet.2006.03.005 16949252 PMC2519033

[B85] Walton M. Rudebeck P. Bannerman D. Rushworth M. (2007). Calculating the cost of acting in frontal cortex. Ann. N.Y. Acad. Sci. 1104, 340–356. 10.1196/annals.1390.009 17360802 PMC2519032

[B86] White N. M. Messier C. Carr G. D. (1987). Operationalizing and measuring the organizing influence of drugs on behavior, in Methods of Measuring the Reinforcing Properties of Abused Drugs, ed Bozarth M. (New York, NY: Springer-Verlag), 591–618.

[B87] Yamamoto R. Ariely D. Chi W. Langleben D. D. Elman I. (2009). Gender differences in the motivational processing of babies are determined by their facial attractiveness. PLoS ONE 4:e6042. 10.1371/journal.pone.0006042 19554100 PMC2698285

