# Supplementary material for: A Quantitative Relationship between Signal Detection in Attention and Approach/Avoidance Behavior
Source: Front Psychol. 2017 Feb 21;8:122. doi: 10.3389/fpsyg.2017.00122 (PMC5318395; doi:10.3389/fpsyg.2017.00122)
Supplement: Supplementary file 1 [file Table1.PDF]

Supplementary Table 1: Power-law mediation of H by K

Model	Model DF	Error DF	RMSE	R	Model F-stat	Model sig.
$H+ = a (K+)^b$	1	181	0.4162	0.6351	122	4.71e-22
**Parameter**	**Estimate**	**t statistic**	**p**			
a	1.381 [1.217, 1.567]	5.05	1.088e-06			
b	0.257 [0.211, 0.303]	11.06	4.707e-22			
**Model**	**Model DF**	**Error DF**	**RMSE**	**R**	**Model F-stat**	**Model sig.**
$H- = a (K-)^b$	1	282	0.2260	0.8339	644	9.05e-75
**Parameter**	**Estimate**	**t statistic**	**p**			
a	1.463 [1.368, 1.565]	11.15	3.768e-24			
b	0.369 [0.340, 0.398]	25.37	9.053e-75			

Legend: 95% confidence intervals are in brackets. RMSE and R are measures of model fit as described in Table 3.
