# Supplementary material for: A Quantitative Relationship between Signal Detection in Attention and Approach/Avoidance Behavior
Source: Front Psychol. 2017 Feb 21;8:122. doi: 10.3389/fpsyg.2017.00122 (PMC5318395; doi:10.3389/fpsyg.2017.00122)
Supplement: Supplementary file 2 [file Table2.PDF]

Supplementary Table 2: Power-law mediation of K by H

Model	Model DF	Error DF	RMSE	R	Model F-stat	Model sig.
$K+ = a\ (H+)^b$	1	181	1.027	0.6351	122	4.71e-22
**Parameter**	**Estimate**	**t statistic**	**p**			
a	2.541 [1.876, 3.441]	6.07	7.479e-09			
b	1.567 [1.288, 1.847]	11.06	4.707e-22			
**Model**	**Model DF**	**Error DF**	**RMSE**	**R**	**Model F-stat**	**Model sig.**
$K- = a\ (H-)^b$	1	282	0.5107	0.8339	644	9.05e-75
**Parameter**	**Estimate**	**t statistic**	**p**			
a	0.942 [0.785, 1.130]	-0.644	0.520			
b	1.884 [1.738, 2.031]	25.37	9.053e-75			

Legend: 95% confidence intervals are in brackets. RMSE and R are measures of model fit as described in Table 3.
