# Supplementary material for: A Quantitative Relationship between Signal Detection in Attention and Approach/Avoidance Behavior
Source: Front Psychol. 2017 Feb 21;8:122. doi: 10.3389/fpsyg.2017.00122 (PMC5318395; doi:10.3389/fpsyg.2017.00122)
Supplement: Supplementary file 3 [file Table3.PDF]

Supplementary Table 3: Power-law mediation of β by H

Model	Model DF	Error DF	RMSE	R	Model F-stat	Model sig.
$\beta = a\,(H+)^b$	1	111	0.5194	0.0696	0.54	0.464
**Parameter**	**Estimate**	**t statistic**	**p**	**q**		
a	2.282 [1.903, 2.737]	9.00	7.056e-15	**--**		
b	0.0610 [-0.103, 0.225]	0.735	0.464	0.138		
**Model**	**Model DF**	**Error DF**	**RMSE**	**R**	**Model F-stat**	**Model sig.**
$\beta = a\,(H-)^b$	1	169	0.5272	0.0246	0.102	0.75
**Parameter**	**Estimate**	**t statistic**	**p**	**q**		
a	2.283 [1.797, 2.899]	6.81	1.617e-10	**--**		
b	-0.0310 [-0.222, 0.160]	-0.320	0.750	0.178		

Legend: 95% confidence intervals are in brackets. RMSE and R are measures of model fit as described in Table 3.
