# Supplementary material for: A Quantitative Relationship between Signal Detection in Attention and Approach/Avoidance Behavior
Source: Front Psychol. 2017 Feb 21;8:122. doi: 10.3389/fpsyg.2017.00122 (PMC5318395; doi:10.3389/fpsyg.2017.00122)
Supplement: Supplementary file 4 [file Table4.PDF]

Supplementary Table 4: Power-law mediation of H by β

Model	Model DF	Error DF	RMSE	R	Model F-stat	Model sig.
$H+ = a\ \beta^b$	1	111	0.5931	0.0696	0.54	0.464
**Parameter**	**Estimate**	**t statistic**	**p**	**q**		
a	2.376 [1.908, 2.957]	7.83	3.139e-12	--		
b	0.0795 [-0.135, 0.294]	0.735	0.464	0.138		
**Model**	**Model DF**	**Error DF**	**RMSE**	**R**	**Model F-stat**	**Model sig.**
$H- = a\ \beta^b$	1	169	0.4186	0.0246	0.102	0.75
**Parameter**	**Estimate**	**t statistic**	**p**	**q**		
a	3.304 [2.947, 3.703]	20.67	4.008e-48	--		
b	-0.0195 [-0.140, 0.101]	-0.320	0.750	0.178		

Legend: 95% confidence intervals are in brackets. RMSE and R are measures of model fit as described in Table 3.
