# Supplementary material for: A Quantitative Relationship between Signal Detection in Attention and Approach/Avoidance Behavior
Source: Front Psychol. 2017 Feb 21;8:122. doi: 10.3389/fpsyg.2017.00122 (PMC5318395; doi:10.3389/fpsyg.2017.00122)
Supplement: Supplementary file 5 [file Table5.PDF]

Supplementary Table 5: Power-law mediation of β by K

Model	Model DF	Error DF	RMSE	R	Model F-stat	Model sig.
$\beta = a\ (K+)^b$	1	136	0.5282	0.0581	0.461	0.498
**Parameter**	**Estimate**	**t statistic**	**p**	**q**		
a	2.271 [1.996, 2.585]	12.53	1.962e-24	--		
b	0.0176 [-0.0337, 0.0690]	0.679	0.498	0.138		
**Model**	**Model DF**	**Error DF**	**RMSE**	**R**	**Model F-stat**	**Model sig.**
$\beta = a\ (K-)^b$	1	179	0.5240	0.1649	5.01	0.0265
**Parameter**	**Estimate**	**t statistic**	**p**	**q**		
a	2.581 [2.223, 2.998]	12.52	3.152e-26	--		
b	-0.0731 [-0.138, -0.0086]	-2.24	0.0265	0.0312		

Legend: 95% confidence intervals are in brackets. RMSE and R are measures of model fit as described in Table 3.
