# Supplementary material for: A Quantitative Relationship between Signal Detection in Attention and Approach/Avoidance Behavior
Source: Front Psychol. 2017 Feb 21;8:122. doi: 10.3389/fpsyg.2017.00122 (PMC5318395; doi:10.3389/fpsyg.2017.00122)
Supplement: Supplementary file 6 [file Table6.PDF]

Supplementary Table 6: Power-law mediation of K by β

**Model**	**Model DF**	**Error DF**	**RMSE**	**R**	**Model F-stat**	**Model sig.**
$K+ = a\ \beta^b$	1	136	1.7419	0.0581	0.461	0.498
**Parameter**	**Estimate**	**t statistic**	**p**	**q**		
a	5.308 [3.035, 9.285]	5.90	2.691e-08	--		
b	0.192 [-0.367, 0.750]	0.679	0.498	0.138		
**Model**	**Model DF**	**Error DF**	**RMSE**	**R**	**Model F-stat**	**Model sig.**
$K- = a\ \beta^b$	1	179	1.1818	0.1649	5.01	0.0265
**Parameter**	**Estimate**	**t statistic**	**p**	**q**		
a	9.845 [7.182, 13.494]	14.31	1.835e-31	--		
b	-0.372 [-0.700, -0.0439]	-2.24	0.0265	0.0312		

Legend: 95% confidence intervals are in brackets. RMSE and R are measures of model fit as described in Table 3.
