# Supplementary material for: A Quantitative Relationship between Signal Detection in Attention and Approach/Avoidance Behavior
Source: Front Psychol. 2017 Feb 21;8:122. doi: 10.3389/fpsyg.2017.00122 (PMC5318395; doi:10.3389/fpsyg.2017.00122)
Supplement: Supplementary file 7 [file Table7.PDF]

Supplementary Table 7: Power-law mediation of d’ by H

**Model**	**Model DF**	**Error DF**	**RMSE**	**R**	**Model F-stat**	**Model sig.**
’ = $a\ (H+)^{b}$	1	111	0.3451	0.0938	0.985	0.323
**Parameter**	**Estimate**	**t statistic**	**p**	**q**		
a	2.489 [2.206, 2.808]	14.98	2.100e-28	**--**		
b	-0.0547 [-0.164, 0.0545]	-0.993	0.323	0.138		
**Model**	**Model DF**	**Error DF**	**RMSE**	**R**	**Model F-stat**	**Model sig.**
’ = $a\ (H-)^{b}$	1	169	0.3378	0.0237	0.0951	0.758
**Parameter**	**Estimate**	**t statistic**	**p**	**q**		
a	2.413 [2.070, 2.813]	11.35	1.505e-22	**--**		
b	-0.0191 [-0.142, 0.103]	-0.308	0.758	0.178		

Legend: 95% confidence intervals are in brackets. RMSE and R are measures of model fit as described in Table 3.
