# Supplementary material for: A Quantitative Relationship between Signal Detection in Attention and Approach/Avoidance Behavior
Source: Front Psychol. 2017 Feb 21;8:122. doi: 10.3389/fpsyg.2017.00122 (PMC5318395; doi:10.3389/fpsyg.2017.00122)
Supplement: Supplementary file 8 [file Table8.PDF]

Supplementary Table 8: Power-law mediation of H by d’

Model	Model DF	Error DF	RMSE	R	Model F-stat	Model sig.
[illegible]$+ = a\,(d')^{b}$	1	111	0.5920	0.0938	0.985	0.323
**Parameter**	**Estimate**	**t statistic**	**p**	**q**		
a	2.927 [2.173, 3.942]	7.15	9.879e-11	--		
b	-0.161 [-0.482, 0.160]	-0.993	0.323	0.138		
**Model**	**Model DF**	**Error DF**	**RMSE**	**R**	**Model F-stat**	**Model sig.**
[illegible]$- = a\,(d')^{b}$	1	169	0.4186	0.0237	0.0951	0.758
**Parameter**	**Estimate**	**t statistic**	**p**	**q**		
a	3.336 [2.805, 3.968]	13.72	2.923e-29	--		
b	-0.0294 [-0.218, 0.159]	-0.308	0.758	0.178		

Legend: 95% confidence intervals are in brackets. RMSE and R are measures of model fit as described in Table 3.
