# Supplementary material for: A Quantitative Relationship between Signal Detection in Attention and Approach/Avoidance Behavior
Source: Front Psychol. 2017 Feb 21;8:122. doi: 10.3389/fpsyg.2017.00122 (PMC5318395; doi:10.3389/fpsyg.2017.00122)
Supplement: Supplementary file 9 [file Table9.PDF]

Supplementary Table 9: Power-law mediation of d’ by K

**Model**	**Model DF**	**Error DF**	**RMSE**	**R**	**Model F-stat**	**Model sig.**
’ = $a\ (K+)^{b}$	1	136	0.3361	0.0687	0.646	0.423
**Parameter**	**Estimate**	**t statistic**	**p**	**q**		
a	2.405 [2.214, 2.611]	21.06	1.197e-44	**--**		
b	-0.0133 [-0.0459, 0.0194]	-0.804	0.423	0.138		
**Model**	**Model DF**	**Error DF**	**RMSE**	**R**	**Model F-stat**	**Model sig.**
’ = $a\ (K-)^{b}$	1	179	0.3332	0.0957	1.65	0.2
**Parameter**	**Estimate**	**t statistic**	**p**	**q**		
a	2.502 [2.275, 2.751]	19.03	7.171e-45	**--**		
b	-0.0267 [-0.0678, 0.0143]	-1.29	0.120	0.0706		

Legend: 95% confidence intervals are in brackets. RMSE and R are measures of model fit as described in Table 3.
