# Supplementary material for: A Quantitative Relationship between Signal Detection in Attention and Approach/Avoidance Behavior
Source: Front Psychol. 2017 Feb 21;8:122. doi: 10.3389/fpsyg.2017.00122 (PMC5318395; doi:10.3389/fpsyg.2017.00122)
Supplement: Supplementary file 10 [file Table10.PDF]

Supplementary Table 10: Power-law mediation of K by d'

Model	Model DF	Error DF	RMSE	R	Model F-stat	Model sig.
[illegible]+ = $a\ (d')^{b}$	1	136	1.7407	0.0687	0.646	0.423
**Parameter**	**Estimate**	**t statistic**	**p**	**q**		
a	8.469 [3.795, 18.901]	5.26	5.388e-07	--		
b	-0.356 [-1.232, 0.520]	-0.804	0.423	0.138		
**Model**	**Model DF**	**Error DF**	**RMSE**	**R**	**Model F-stat**	**Model sig.**
[illegible]- = $a\ (d')^{b}$	1	179	1.1927	0.0957	1.65	0.2
**Parameter**	**Estimate**	**t statistic**	**p**	**q**		
a	9.818 [6.036, 15.969]	9.27	6.117e-17	--		
b	-0.343 [-0.868, 0.183]	-1.286	0.120	0.0706		

Legend: 95% confidence intervals are in brackets. RMSE and R are measures of model fit as described in Table 3.
