# Supplementary material for: A Quantitative Relationship between Signal Detection in Attention and Approach/Avoidance Behavior
Source: Front Psychol. 2017 Feb 21;8:122. doi: 10.3389/fpsyg.2017.00122 (PMC5318395; doi:10.3389/fpsyg.2017.00122)
Supplement: Supplementary file 11 [file Table11.PDF]

Supplementary Table 11: Fits for different models of the form H = f(K, d')

		Logarithmic: $H = a + b \cdot \ln(d') + c \cdot \ln(K)$	Power Law Multiplicative: $H = a * (d')^{b} * (K)^{c}$	Power Law Additive: $H = a + (d')^{b} + K^{c}$
**Approach (H+)**	**Parameter**	**Estimate**	**Estimate**	**Estimate**
Intercept/scaling constant	a	0.920	1.099	-1.072
		[0.474, 1.367]	[0.847, 1.350]	[-1.497, -0.646]
		t(135) =4.08	t(135) = 8.64	t(135) = -4.98
		p =7.64e-5	p = 1.41e-14	p = 1.89e-6
d'	b	0.217	0.046	0.190
		[-0.228, 0.662]	[-0.120, 0.212]	[-0.159, 0.539]
		t(135) = 0.964	t(135) = 0.547	t(135) = 1.08
		p = 0.337<br>q = 0.134	p = 0.585<br>q = 0.149	p = 0.283<br>q = 0.134
K^{+}	c	0.675	0.323	0.352
		[0.590, 0.761]	[0.271, 0.375]	[0.327, 0.377]
		t(135) = 15.55	t(135) = 12.31	t(135) = 27.67
		p = 6.82e-32	p = 8.08e-24	p = 1.72e-57
RMSE		.8817	.8753	.8778
R		.8011	.8043	.8031
Model F-stat		F(2,135) = 121	F(2,135) = 412	F(2,135) = 123
Model sig.		p = 7.98e-31	p = 9.60e-68	p = 4.45e-31
**Avoidance (H-)**	**Parameter**	**Estimate**	**Estimate**	**Estimate**
Intercept/scaling constant	a	1.318	1.546	-0.484
		[1.060, 1.576]	[1.368, 1.723]	[-0.768, -0.200]
		t(178) = 10.09	t(178) = 17.15	t(178) = -3.36
		p =3.31e-19	p = 1.46e-39	p = 9.55e-4
d'	b	0.107	0.026	0.089
		[-0.123, 0.337]	[-0.047, 0.100]	[-0.147, 0.325]
		t(178) = 0.915	t(178) = 0.711	t(178) = 0.745
		p = 0.361<br>q = 0.134	p = 0.478<br>q = 0.134	p = 0.457<br>q = 0.134
K^{-}	c	0.939	0.336	0.441
		[0.875, 1.004]	[0.301, 0.370]	[0.421, 0.461]
		t(178) = 28.83	t(178) = 19.08	t(178) = 42.90
		p = 5.58e-69	p = 6.72e-45	p = 8.83e-96
RMSE		.5198	.5702	.5976
R		.9079	.8880	.8762
Model F-stat		F(2,178) = 417	F(2,178) = 2,210	F(2,178) = 294
Model sig.		p = 6.28e-68	p = 1.23e-140	p = 3.82e-57

Legend: 95% confidence intervals are in brackets. RMSE and R are measures of model fit as described in Table 3.
