# Supplementary material for: A Quantitative Relationship between Signal Detection in Attention and Approach/Avoidance Behavior
Source: Front Psychol. 2017 Feb 21;8:122. doi: 10.3389/fpsyg.2017.00122 (PMC5318395; doi:10.3389/fpsyg.2017.00122)
Supplement: Supplementary file 12 [file DataSheet1.DOCX]

**Supplemental Information** (Viswanathan et al. “A quantitative interaction between signal detection in attention and approach/avoidance behavior”)

*For the MGH Phenotype Genotype Project in Addiction and Mood Disorder (PGP), data collection for each subject included the following:*

Counterbalancing was performed across subjects in three ways for this study. First, the order of performing 1.5T or 3.0T scanning was counterbalanced across subjects, with all scanning performed at the Martinos Center for Biomedical Imaging, Massachusetts General Hospital and Harvard Medical School. Second, the order of functional MRI scans on the 3.0T scanner was counterbalanced across subjects. Third, the order of the post-scanning testing was counterbalanced across tests related to material used in the scan sessions; material collected post-scanning that was not related to the scanning was always collected after the post-scan assessments, and further randomized across themselves.

*A. Clinical phenotyping included*:

1. Medical history/Review of symptoms (ROS) and medication status,
2. Developmental, demographic data and Hollingshead assessment for matching socioeconomic variables between cohorts,
3. SCID-I interview (DSM-IVR axis I),
4. An ASAM interview (addiction),
5. Blood and urinary analysis: Quest Labs Chem 26 Fe, CBC Hematogram with platelets/differential, Thyroid Stimulating Hormone, HIV-1 with reflex western, and HepC. Blood was also be collected for:1) carbohydrate deficient transferrin as a marker for chronic alcohol use; 2) cotinine-nicotine metabolite, 3) homocysteine, C-reactive protein, and 4) lipid/cholesterol differential (cardiovascular disease that would affect brain functions). Urine via MGH toxicology screen,
6. Hair analysis for short- and long-term drug use (in the cocaine dependent subjects and their first-degree relatives only) (Avitar labs; drug history for 90 days)
7. Urine tox on day of scan,
8. Time-line follow-back assessment for 3 months of menstrual history in women, plus urine surge assessment on the day of scan
9. Edinburgh Handedness assay, Anger Attack Questionnaire (AAQ), Hamiltion Rating Scale for Depression (HAM-D), Inventory of Depressive Symptomatology (Self -Report) (IDS-SR) , MGH Cognitive Physical Functioning Questionnaire, Short Form (36) Health Survey, Symptom Questionnaire (SQ), Temperament and Character Inventory (TCI) , Wechsler Adult Intelligence Scale-Revised (WAIS-R), and TOVA standardized attention performance task,
10. ECG by senior cardiologist,
11. Blood draws for DNA and EBV lymphoblast cell lines.

*B. Neuroimaging phenotyping included scanning on 3T and 1.5T Siemens systems*:

All subjects scanned with bitebars to minimize motion and with use of MRI-compatible glasses to normalize vision if needed. Subjects were counterbalanced with regard to being scanned on the 3T Siemens TRIO or the 1.5T Siemens Avanto system first.

3T: All fMRI scans in (1a-e) were counterbalanced across subjects to mitigate the order of exposure to any one stimulus. fMRI scanning followed acquisition of one high res structural scan.

1. The five published fMRI paradigms were ones that have had variants run at other centers with similar results:

(a) monetary expectancy and outcomes (gains and losses) based on prospect theory (see Breiter et al., 2001)

(b) facial beauty/reward (based on Aharon et al., 2001); Note: this task was immediately followed by a quick questionnaire about which faces were seen, and their characteristics,

(c) thermal pain (based on Becerra et al., 2001),

(d) assessment of communication of motivational intent in others (Ekman facial emotion) (based on Breiter et al., 1996); Note: this task was immediately followed by a quick questionnaire about which faces were seen, and their characteristics,

(e) continuous performance task - attention during different probability conditions between a cue and its predicted target (based on Seidman et al., 1997; Breiter & Rosen, 1999),

These scans were performed after the fMRI tasks:

1. Resting quantitative regional blood perfusion (CASL),
2. Regionalized white matter connectivity (DTI),
3. High-resolution structural scan at the end (same sequence as at the beginning).

1.5T: The 1.5T structural scans (1) – (3) were performed in a fixed order.

1) 15-min high resolution T1 scan for CMA anatomist-guided segmentation

2) 3 MPRAGE scans for averaging to be used with Freesurfer

3) Proton density, and T2 relaxometry

*C. Offline Cognitive Tests*:

Per the list presented below, the Keypress Speed test was done twice, just before and just after completion of the Keypress tests in (9) and (10). The Keypress Practice was done just before the Keypress tests. The Rating Practice was done before the Rating tests. The sequence of tests (1), (2), (6,7), (9,10) were counterbalanced across subjects, and the sequence of (6,7) and (9,10) were further counterbalanced across subjects. Tests (12) – (15) were always at the end of the offline testing session, and their sequence relative to each other was randomized across subjects.

1) Heat paradigm post-questionnaire*

2) Spinner paradigm post-questionnaire (re: how much money won, and feelings during task conditions)*

3) Keypress Speed Practice*

4) Keypress Speed test (first) *

5) Rating Practice (the same questions for Ekman and Beauty Ratings: “Rate how you feel about each face? -10 is most likely to hurt/punish/disappoint you, and +10 is most likely to help/reward/please you”)

6) Ekman face Rating

7) Beauty face Rating

8) Keypress Practice *

9) Ekman face Keypress to alter time of viewing

10) Beauty face Keypress to alter time of viewing

11) Keypress Speed test (second) *

12) Final PQ *

13) Paper questionnaire *

14) Positive and Negative Affect Schedule (PANAS)

15) Spielberger State Trait Anxiety Inventory (STAI)

* Technician present during this offline task to answer questions.
